# Comparative transcriptome analysis of bovine blastocysts reveals specific effects of the oocyte source and the environments during maturation and early embryo development

**DOI:** 10.1186/s12864-025-11848-8

**Published:** 2025-07-17

**Authors:** Mohammad Bozlur Rahman, Eva Held-Hoelker, Mohammed Saeed-Zidane, Franca Rings, Dessie Salilew-Wondim, Dawit Tesfaye, Ahmed Gad, Samuel Gebremedhn, Ernst Tholen, Karl Schellander, Christine Große-Brinkhaus, Michael Hoelker

**Affiliations:** 1 Institute of Animal Sciences, Animal Breeding, University of Bonn, Endenicher Allee 15, Bonn, 53115 Germany; 2 Department of Animal Science, Biotechnology and Reproduction of farm animals, University of Goettingen, Burckhardtweg 2, Göttingen, 37077 Germany; 3 Institute of Animal Breeding and Husbandry, Molecular Genetics Group, Christian-Albrechts-University Kiel, Kiel, 24118 Germany; 4 Department of Biomedical Sciences, Animal Reproduction and Biotechnology Laboratory, Colorado State University, 3105 Rampart Rd, Fort Collins, CO 80521 USA

**Keywords:** IFOT, Gene expression, Embryo source, Developmental environment

## Abstract

**Background:**

Although differences between in vivo and in vitro derived bovine blastocysts in terms of global gene expression profiles have been reported, comparative transcriptome analyses specifically addressing the sustained impact of the oocyte source or the in vitro culture environments during maturation and post-maturation period are remain limited. Therefore, the present study aimed to investigate the specific impacts of oocyte origin and the culture environment during and after maturation on the gene expression signature at blastocyst stage. To achieve this, we utilized our recently developed technique that enables intrafollicular transfer of immature and matured slaughterhouse-derived oocytes into dominant or preovulatory follicles.

**Results:**

The presents study identified a total of 1052 differentially expressed genes between in vitro and in vivo derived blastocysts, many of which are involved in key pathways related to protein synthesis, protein degradation and cell-cycle regulation. The majority of these genes (*n* = 913), particularly those associated with “ubiquitin mediated proteolysis”, “proteasome activity” as well as “cell cycle” related pathways, were differentially expressed due to the in vitro environment following oocyte maturation. Moreover, a distinct set of genes (*n* = 109) including *DHCR7*, *DHCR24*, *HMGCR*, *HMGCS1* and *SCD5*, which are crucial for cholesterol biosynthesis and lipid metabolism, were altered in response to the in vitro environment during oocyte maturation. Notably, the origin of the immature oocyte also appeared to predetermine the later expression outline of a set of genes (*n* = 28), including *DLD* and *PLAC8*, which are implicated in implantation success and calf delivery.

**Conclusions:**

The present study provides a comprehensive overview of transcriptomic alterations and pathway disruptions resulting from the in vitro environment following oocyte maturation, offering insight into potential mechanisms underlying embryonic genome activation, DNA duplication and appropriate cell cleavage. The differential expression of genes involved in cholesterol biosynthesis and lipid metabolism due to the in vitro maturation environment may contribute to the reduced cryotolerance observed in the resulting blastocysts. Furthermore, dysregulation of specific genes as a consequence of oocyte source has implications for post-implantation developmental competence. Collectively, these findings advance our understanding of the molecular determinants affecting embryonic developmental potential. The expression signature of these pathways could therefore be used to assess the impact of various treatments and culture environments on embryonic development. In addition, the insights gained from this study could inform future strategies to improve the quality of embryos in in *vitro* production systems through the targeted modulation, either enhancement or inhibition, of specific genes or pathways.

**Supplementary Information:**

The online version contains supplementary material available at 10.1186/s12864-025-11848-8.

## Background

Since the inception of the in vitro embryo production system, numerous efforts have been made to improve their efficiencies, leading to a significant improvement over the last decades. However, in vitro produced embryos still lag behind their in vivo counterparts in terms of developmental competence as well as quality and viability of the resulting blastocysts [1]. In particular, in vitro culture conditions have been reported to exert detrimental effects on early embryonic development and/or quality [2], thereby resulting incomplete fulfillment of the promise of this technique [3]. Our group has extensively investigated bovine blastocysts by culturing embryos in alternative environments (in vitro vs. in vivo) around the time of embryonic genome activation [4]. In that study we were able to demonstrate that in vitro culture conditions critically alter the transcriptome profile of subsequent blastocysts in stage specific manner. Likewise, specific culture conditions, such as supplementation of serum the in vitro culture media, have been associated with altered transcriptomic and epigenetic profiles, which may contribute to fetal overgrowth in ruminants, a condition commonly known as Large Offspring Syndrome (LOS) [5, 6]. In this regards, Wydooghe and colleagues reported that the transcriptome profile of blastocysts produced in serum-free culture conditions were more close to in vivo derived blastocysts compared to those cultured in presence of serum [7].

In order to unmask the limitations of the in vitro culture environment, the essential role of the oviduct representing the physiological environment during early embryo development has therefore been investigated in many laboratories across the world. More specifically, murine [8, 9], rabbit [10, 11], sheep [12, 13] as well as bovine oviducts [14, 15] have been used as a intermediate hosts for bovine embryos to allow development within the physiological or at least within a nearly physiological environment provided by a homologous or heterologous oviduct. In this context, and to avoid surgical interventions, early *in vitro-*produced bovine embryos have been successfully transferred endoscopically into the oviducts of live heifers and cows [16–18]. These studies clearly demonstrated that the transfer of *in vitro-*fertilized bovine zygotes into the oviduct resulted in the development to blastocysts with enhanced cryotolerance [19] as well as higher developmental capacity after transfer to recipients [1]. Particularly, these studies highlighted that the postfertilization culture environment had a significant impact on the quality of the resulting blastocysts, especially in terms of their global gene expression profiles [20]. Therefore, these findings further underscore that the transfer of early IVP derived zygotes into the oviduct provides a valuable model for elucidating the critical role of the embryonic environment during development up to the blastocyst stage. However, endoscopic intrafallopian transfer of bovine embryos remains a technically challenging procedure that demands a high level of skills. Because this technique allows for the transfer of embryos into the oviduct but not oocytes into the pre-ovulatory follicle, it cannot be used to assess the influence of the developmental environment during oocyte maturation or the impact of oocyte selection prior to final maturation, i.e. the effect of oocyte source. Thus, the effects of the environment during maturation and fertilization for the later quality at the blastocyst stage are largely unresolved. To address this research gap, specifically, to elucidate the distinct contributions of oocyte source prior to final maturation and the developmental environment during maturation, fertilization, and culture, the present study employed a fundamentally novel approach. We utilized a recently developed technique, intrafollicular transfer of in vitro matured abattoir-derived oocytes (IFOT-M), which enables the direct transfer of in vitro matured oocytes into the preovulatory follicle under ultrasound guidance. That strategy allowed subsequent fertilization within the oviduct following artificial insemination [21] and enabled the collection of large number of blastocysts developed within the physiological environment after maturation. Notably, the quality of these IFOT-M derived blastocysts closely resembled fully in vivo derived blastocysts in terms of lipid content and cryotolerance. Furthermore, the transfer of these frozen-thawed IFOT-M derived embryos resulted in viable pregnancies and the birth of healthy calves [21]. However, the pregnancy rates remained lower in IFOT-M derived embryos compared to fully in vivo derived embryos, highlighting the need for further investigation into the underlying molecular mechanisms and pathways that differentiate these two groups. In another study, IFOT was also conducted using immature slaughterhouse derived bovine oocytes which also resulted in healthy offspring [22]. Of high importance, intrafollicular transfer of immature bovine oocytes (IFOT-IM) allows maturation within the follicle representing the physiological environment of the maturing oocyte as well as fertilization and development within the fallopian tube and the oviduct [22]. IFOT-IM and IFOT-M embryos thus represent recognised models for distinct phenotypes. Apart from the proof of principle that intrafollicular transfer of immature oocytes into preovulatory follicles is a feasible technique resulting in birth of healthy calves, that technique also allowed for the first time full in vivo development of immature bovine oocytes collected from slaughterhouse ovaries. With other words, that technique allows for the first time to compare the impact of the source of the immature oocyte prior final maturation for the later embryo quality at the blastocyst stage. Although differences between in vivo and in vitro derived bovine blastocysts in terms of gene expression profile have reported previously [23] it has to be noted, that in vivo derived embryos are usually obtained by superovulation. This also raises the question of whether the differences between in vivo and in vitro derived embryos could be due to the super stimulation treatment of the in vivo derived embryos, which could have lasting effects on the gene expression profile of the subsequent blastocyst. Therefore, the aim of the present study was to investigate the specific impact of the oocyte source as well as the implications of contrasting environments specifically during and after maturation, namely fertilization and early preimplantation development, in terms of the global gene expression profile. In addition, we aimed to uncover the specific molecular mechanisms and pathways affected by these distinct differentially expressed genes, which are particularly influenced by the oocyte source and the in vitro environment during maturation, fertilisation and early development up to the blastocyst stage, as a model for adverse environmental conditions. In order to elucidate the molecular pathways and regulatory mechanisms underlying the differences in characteristics, quality and viability of bovine embryos arising from distinct origins and developmental environments, we specifically analyzed IFOT-IM and IFOT-M embryos, recognized models representing contrasting phenotypes and developmental potential. Their transcriptomic profiles were compared to fully in vitro derived and fully in vivo-derived bovine embryos. We therefore took advantage of the IFOT-M and IFOT-IM techniques to transfer abattoir-derived immature or in vitro matured oocytes into dominant or preovulatory follicles to allow subsequent in vivo development to the blastocyst stage prior to investigating the global gene expression landscape. Addressing these questions is expected to significantly enhance our understanding of the limitations of in vitro embryo development, particularly those arising from oocyte source and environment-induced dysregulation of early embryonic molecular pathways. Gaining insight into these underlying causes may enable the development of targeted strategies to modulate the expression of key pathways, thereby improving assisted reproductive technologies (ART) for enhanced early embryo development. Moreover, these findings may offer valuable insights applicable to human assisted reproduction.

## Methods

### Experimental design

**Fig. 1:**
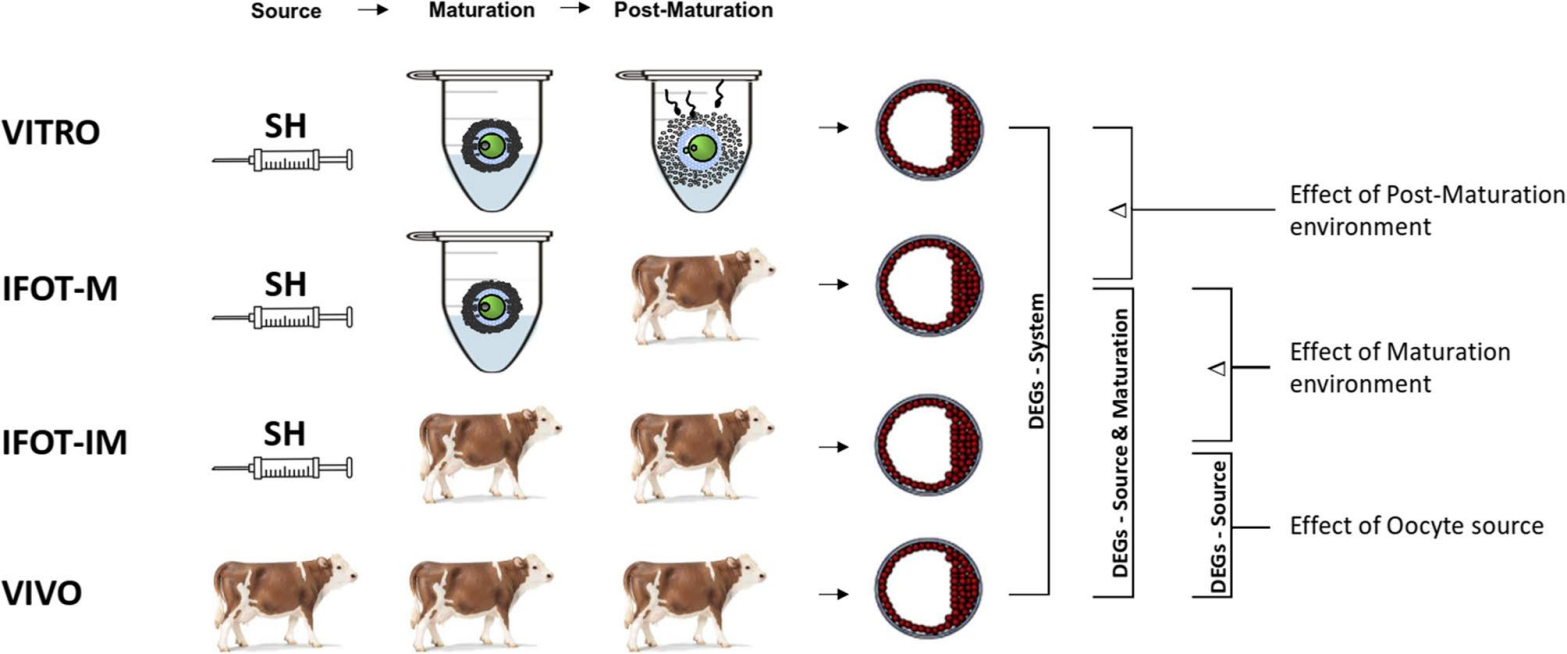
Experimental outline of the study comparing 4 contrasting embryo groups. (VITRO) Embryos developed from immature oocytes of slaughterhouse (SH) ovaries in vitro (IFOT-M), Embryos developed from immature oocytes of slaughterhouse ovaries after transfer into a preovulatory follicle after in vitro maturation to allow in vivo fertilization and in vivo development, (IFOT-IM) Embryos developed from immature oocytes of slaughterhouse ovaries transferred into a dominant follicle to allow in vivo maturation, in vivo fertilization and in vivo development. (VIVO) In vivo derived embryos. Differentially expressed genes (DEGs) between VITRO and VIVO are considered to be modulated by the production system. DEGs between VITRO and VIVO groups (system effect) minus DEGs between IFOT-M and VIVO (effect of source or maturation environment) are considered to be modulated by the post-maturation environment. DEGs between IFOT-M and VIVO minus DEGs between IFOT-IM and VIVO groups (effect of the oocyte source) are considered to be modulated by the maturation environment. DEGs between IFOT-IM and VIVO are considered to be modulated due to the oocyte source

In this study, the transcriptome profile of expanded bovine blastocysts produced in three different experimental conditions, namely (I) VITRO (effect of the system), (II) IFOT-M (Intrafollicular transfer of in vitro matured oocytes into preovulatory follicles) and (III) IFOT-IM (Intrafollicular transfer of immature oocytes into dominant follicles) were compared with in vivo derived blastocysts (IV, VIVO) serving as control. An overview about the contrasting embryo groups with the respective oocyte source as well as the subsequent environments during development is provided by Fig. 1. To determine the genes whose expression is specifically influenced by the developmental environment after maturation, DEGs between the VITRO and VIVO groups (system effect) are reduced by those that also show different expression when comparing the IFOT-M vs. VIVO groups (effect of Source or Maturation environment). To determine the genes whose expression is specifically influenced by the developmental environment during maturation, DEGs between the IFOT-M and VIVO groups are reduced by those that simultaneously show different expression when comparing the IFOT-IM vs. VIVO groups (Effect of the oocyte source). Finally, the genes whose expression is specifically influenced by the oocyte source are determined by comparing the IFOT-IM and VIVO groups. Animals used in this study were treated following the guidelines of the Society of Reproduction an experimental design was approved by the Federal Ministry of Food and Agriculture in Germany (REF-Nr. 84-02.04.2014.A499 and REF-NR. 84-02.04.2014.A500). To determine the transcriptome profiles, molecular signatures and affected pathways, pools of 15 expanded blastocysts were analyzed by Affymetrix GeneChip^®^ Bovine 1.0 ST array. Three independent biological replicates were investigated for each of the four experimental groups.

### In vitro maturation of oocytes

In vitro oocyte maturation was performed as described by Kassens and colleagues [21]. In brief, bovine ovaries were obtained from a local slaughterhouse and brought to the lab in physiological saline (0.9% NaCl) at 30 °C within 3 h of slaughter of cows. Cumulus oocyte complexes (COCs) were aspirated from small follicles (2–8 mm), and COCs with a homogenous, evenly granulated ooplasm, with oocytes surrounded by at least three layers of cumulus cells, were transferred to modified Tissue Culture Medium 199 (TCM; Sigma) supplemented with 4.4 mM HEPES, 33.9 mM NaHCO3, 2 mM pyruvate, 2.9 mM calcium lactate, 55 µg ml^−1^ gentamycin and 12% heat-inactivated ECS. After washing three times, COCs were cultured in groups of 50 in 400 µl of modified TCM supplemented with 10 µg ml^−1^ FSH (FSH-p; Schering-Plough) at 39 °C in a humidified atmosphere with 5% CO_2_ in air. In vitro maturation of oocytes was started through coinciding the time that recipients have received the final gonadotropin releasing hormone (GnRH) injection and in vitro maturation process lasted for 16–22 h until the oocytes were transferred into a preovulatory follicle (IFOT-M) or oocytes were taken for full in vitro development (VITRO) alternatively.

### Intrafollicular oocyte transfer of in vitro matured oocytes (IFOT-M)

Intrafollicular transfer in this study was performed with in vitro matured oocytes (IFOT-M) as described in detail in one of our previous studies by Kassens and colleagues [21]. Briefly, intrafollicular transfer of in vitro matured oocytes (IFOT-M) into the preovulatory follicle was performed 16–22 h after the last GnRH injection. The day of IFOT-M was considered as day 0. Heifers were artificially inseminated with fresh semen (50 million spermatozoa per dose) from the same bull used for in vitro embryo production and superovulation/flushing. The detailed procedure is described in our previous paper [21]. Briefly, the system for transferring the oocytes to the preovulatory or dominant follicles consisted of an oocyte pick up (OPU) device. However, instead of a pump to create negative pressure, the OPU line was connected to a syringe to fill the system with PBS and a laboratory pipette to create positive or negative pressure (Supplementary Fig. 1). For transfer into the follicle, in vitro matured oocytes were loaded into the tip of the OPU system, represented by a disposable single-lumen needle (0.45 × 22 mm; G26; Sterican). A maximum volume of 200 µl of PBS containing 50 oocytes was aspirated. The needle was then carefully inserted through the vaginal wall and peritoneum while observing the preovulatory follicle on the monitor. Care was taken to orient the needle and the ovary so that it passed through as much of the ovarian stroma as possible before reaching the follicle, in order to avoid follicle leakage. When the needle tip was successfully positioned in the antrum near the center of the follicle, the PBS-containing oocytes were delivered into the antrum (Supplementary Fig. 2). IFOT-M-derived embryos were then collected by embryo flushing on day 7, as described in detail below.

### Intrafollicular oocyte transfer of immature oocytes (IFOT-IM)

Intrafollicular transfer in this study was performed with immature oocytes (IFOT-IM) as described in detail in one of our previous studies by Kassens and colleagues [21]. Briefly, intrafollicular transfer of immature oocytes was performed in the dominant follicles of synchronised Simmental heifers at the time of the last GnRH injection. The day of IFOT-IM was considered as day − 1. To coincide with the time of IFOT-M, immature oocytes were expected to be retained in the follicle for at least 16–22 h prior to ovulation. Immediately prior to IFOT-IM, 16 h after the last GnRH injection, heifers were artificially inseminated with fresh semen (50 million spermatozoa per dose) using the same bull as for IFOT-M, in vitro embryo production and superovulation/flushing. The detailed procedures are available in our previous paper [21] and were the same as those previously described for in vitro matured oocytes except that immature cumulus oocyte complexes were transferred into the dominant follicle instead of in vitro matured oocytes into the preovulatory follicle. IFOT-IM-derived embryos were then collected on day 7 by embryo flushing as described in detail below.

### In vitro fertilization (IVF) and in vitro embryo production (IVP)

In vitro fertilization of in vitro matured oocytes as well as in vitro culture of presumptive zygotes was performed as described earlier by Kassens and colleagues [21]. Briefly, in vitro fertilization of in vitro matured oocytes was performed in Fert-TALP medium supplemented with 20 µM penicillamine, 10 µM hypotaurine, 2 µM epinephrine, 6 mg/ml BSA-*FFA* (Fatty Acid Free BSA), 50 µg/ml gentamycin, and 10 µg/ml heparin. For IVF, semen of the same sire (red Holstein Friesian genotype) as used for the generation of IFOT-IM, IFOT-M and VIVO embryos was used. Final concentration of sperm in fertilization droplets was adjusted to 2 × 10^6^ sperm/ml. Following 18 h of co-incubation, the presumptive zygotes were vortexed to remove cumulus cells and excess sperm. Then the zygotes were washed three times and transferred to in vitro culture. Embryo culture was performed in groups of 50 with 5% CO_2_ in air at 39 °C for up to 7 days in 400 µl of SOFaa supplemented with 0.4% BSA-*FFA* overlaid with mineral oil.

### Superovulation protocol

The detailed protocol for superovulation has already been reported in an earlier study of Kassens and colleagues [21]. In brief, Simmental heifers (~ 400 kg) were synchronization by administration of 500 µg of cloprostenol, i.m. (Estrumate, Essex Tierarznei, Germany) 11 days apart. Forty-eight hours after each of the cloprostenol injections, cows received 20 µg of GnRH (Receptal; Intervet, Boxmeer, The Netherlands). Superovulation treatment was started after 12 days of the last GnRH administration. Accordingly, cows received the first of 8 consecutive administrations of FSH during the course of 4 days in decreasing dosages (in total, 300–400 mg of FSH; Stimufol, University of Liege). At 60 and 72 h after the initial administration of FSH, cows received two doses of cloprostenol. Finally, 48 h after the first of the two cloprostenol injections, ovulation was induced by 20 µg of GnRH and immediately after GnRH injection heifers received a first dose artificial insemination. Artificial insemination in the same recipient was repeated twice within a 12 h interval (i.e., every cow received 3 consecutive inseminations).

### Embryo flushing

The procedures for collecting in vivo derived blastocyst have already been reported in an earlier study of Kassens and colleagues [21]. Briefly, seven days after the IFOT procedure or superovulation treatment (day of heat for IFOT or day of AI for superovulation was defined as Day 0), embryos were flushed from the uterus of recipient cows. An embryo-flushing catheter (CH15; Wörrlein) was fixed in the uterine horns. Afterwards, the embryos were flushed out by washing each uterine horn with 500 ml of PBS. In this, regards, the uterus was flushed using a catheter connected with an embryo filter (Emcon filter 1; no. 04135; Immuno Systems Inc.). Immediately after flushing, embryos were washed twice in PBS and were evaluated by developmental stages. To calculate the real developmental rates of IFOT-derived embryos, the corresponding flushed embryos were subtracted by the recipient’s own embryo. Consequently, the embryo at the highest stage of development was subtracted for each IFOT recipient to calculate accurate quantities of embryos flushed out in excess. The subtracted embryos were thought to be derived from the respective recipient’s oocytes. Therefore, it can be assumed that the chances of false positive inclusion of recipient oocyte-derived embryos as of IFOT-derived embryos were very low. Nevertheless, a potential limitation of this study is the possibility that the IFOT-IM and IFOT-M embryo pools may have included a small number of embryos with gene expression profiles resembling those of fully in vivo-derived embryos. In the context of our transcriptomic analysis, this would likely result in a conservative bias - meaning that the observed differences between the IFOT-M/IFOT-IM groups and the VIVO group may be smaller than they are in reality. Therefore, the molecular distinctions reported in this study are likely underestimated.

### Embryo storage and RNA extraction

The quality of blastocysts was assessed according to International Embryo Transfer Society (IETS) standards by the same trained individual. Only expanded blastocysts of quality 1 were selected for this study. The blastocysts were washed three times in RNase-free PBS (Phosphate buffered saline; Ambion), placed in pools of 5 blastocysts in 2 µL of lysis buffer having RNase inhibitor and immediately stored at − 80 °C. In each group, 3 pools of 5 expanded blastocysts were combined to form a pool of 15 expanded blastocysts, providing the required amount of total RNA for Affymetrix Array without introducing amplification bias. Total RNA was extracted using the Single Cell RNA Purification Kit (Norgen, P/N 51800) according to the manufacturer’s instructions. On-column DNA digestion was done by using RNase-free DNase (Qiagen GmbH, Hilden, Germany). RNA was eluted in 18 µl of elution buffer and the initial quantity and purity were measured by NanoDrop^®^ spectrophotometry (ND-8000; NanoDrop Technologies). Later, the RNA integrity was verified on an Agilent 2100 Bioanalyzer (Agilent Technologies Inc., Santa Clara, CA) using RNA 6000 Pico Kit. The RIN values of ≥ 7.0 with rRNA ratio of ≥ 1.5 were considered for microarray target preparation and hybridization (Additional File 1). RNA samples were stored at − 80 °C until use.

### Microarray target preparation and hybridization

Microarray hybridization was adopted from a publication [24]. Briefly, 15 ng total RNA samples from each of the biological replicates were processed with the GeneChip^®^ WT Pico Reagent Kit (P/N 902622; Affymetrix Inc., Santa Clara, CA, USA) in order to prepare the target probes of 12 microarrays according to the manufacturer’s instructions. In brief, the total RNA was subjected to synthesize the first-strand cDNA containing a T7 promoter sequence at the 5′ end followed by synthesis of double-strand cDNA using DNA polymerase with the presence of RNase H. Then, the double-stranded cDNA was subjected to in vitro transcription by T7 RNA polymerase for synthesis of the antisense RNA (complementary RNA, cRNA). The cRNA preparation was then purified using purification beads to improve its stability. From 20 µg of purified cRNA, the sense-strand cDNA (ss-cDNA) was synthesized by reverse transcription. The ss-cDNA contained dUTP at a fixed ratio relative to dTTP and the remaining cRNA was degraded by RNase H. Following purification and quantification, 5.5 µg of ss-cDNA in a 46 µl volume was fragmented by uracil-DNA glycosylase (UDG) and apyrimidinic endonuclease 1 (APE 1) at the unnatural dUTP residues and breaks the DNA strand. The fragmented ss-cDNA was then labeled with terminal deoxynucleotidyl transferase (TdT) using the biotin-linked labeling reagent. The hybridization of microarray probes followed by washing and staining was performed with the GeneChip^®^ Hybridization, Wash and Stain Kit (P/N 900720-C, Affymetrix Inc., Santa Clara, CA). For hybridization, about 130 µl of cocktail containing at least 3.5 µg of biotinylated ss-cDNA probes were injected into the GeneChip^®^ Bovine Gene 1.0 ST Array (P/N 901921, Affymetrix Inc., Santa Clara, CA, USA) and incubated for 16 h in a hybridization oven (Gene-Chip^®^ Hybridization oven 640; Affymetrix Inc.) at 45 °C with 60 rpm. The hybridized chips were stained and washed in a fluid station (GeneChip^®^ Fluidics Station 450; Affymetrix Inc.) and scanned by Affymetrix GeneChip^®^ scanner 3000 7G. The Affymetrix GeneChip^®^ Command Console™ (AGCC) software was used to evaluate the array images and to export the spot intensity data in the CEL file format.

### Microarray data analysis

Microarray data analysis was adopted form an earlier publication of us by Islam and colleagues [24]. Briefly, pre-processing of microarray raw dataset was performed using the Affymetrix Expression Console software (v1.0) [25]. The RMA (Robust Multi-Array Average) based background correction and quantile normalization of microarray data were performed at transcript level. For quality control, some quality plots of the raw intensity data were checked before and after the normalization. In total 12 arrays were used for further analyses. After normalization, the main probes (26,774) of the array were extracted followed by about 2359 low expressed probes were filtered out. Finally, expression dataset comprising 24,415 transcript probes were analyzed by Affymetrix Transcription Analysis Console software (TAC v.3.0) [26], and subjected for downstream analyses. Probe to transcript level annotation was performed following Affymetrix annotation algorithm [26]. The raw and processed data generated during this study are stored in the NCBI repository: https://www.ncbi.nlm.nih.gov/geo/query/acc.cgi?acc=GSE284466.

### Hierarchical clustering

Hierarchical clustering was performed with RMA normalized transcripts differentially expressed among the target groups (FC > 2, raw *p*-value < 0.05, False discovery rate (FDR) < 0.1) using the Affymetrix TAC software.

### Functional annotation & sub-network enrichment analysis of genes

Ensembl gene IDs of the differentially expressed genes were further analyzed with the ClueGO v2.5.2 plugin of the Cytoscape 3.7.0 software [27, 28]. The Cytoscape plugin ClueGO was used to export the significantly enriched gene ontologies (levels 3–5) related to cellular component, biological process, molecular function and KEGG pathway (FC > 2, Bonferroni corrected *p*-value < 0.01). Sub-networks were generated representing the expression outline of the common genes shared by the significantly enriched KEGG pathways.

### Construction of the protein–protein interaction networks

For statistical, visual and network-based analysis of Protein-Protein Interactions (PPI) of target genes, a sub-network analysis was performed using the NetworkAnalyst online tool (https://www.networkanalyst.ca) [29] using STRING database. Depending on the number of target genes and interactions, confidence score for PPI analysis was set either to high (900) or medium (400). The *p*-values of the networks were calculated based on their connectivity assuming null hypothesis that there is no difference between the number of “internal” and “external” connections to a particular node in the module with *p* < 0.05 was considered as statistically significant.

## Results

### Developmental metrics and phenotype of contrasting experimental groups

**Fig. 2:**
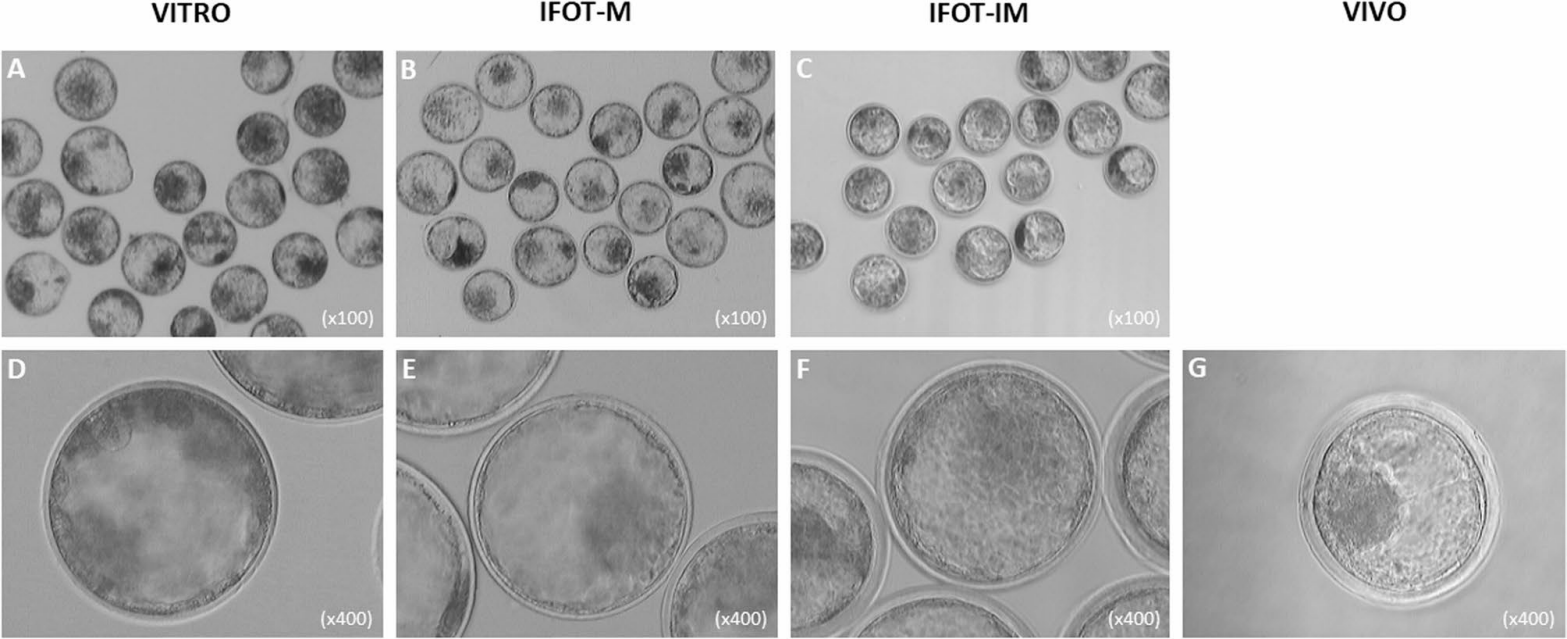
Representative imagines of pools of VITRO (**A**), IFOT-M (**B**) and IFOT-IM (**C**) embryos (x100 magnification) as well as individual blastocysts of VITRO (**D**), IFOT-M (**E**), IFOT-IM (**F**) and VIVO (**G**) embryos (x400 magnification) demonstrating that VITRO embryos appeared opaquer compared to all other groups developed within the oviduct after cleavage (VITRO-M) of after maturation (VITRO-IM & VIVO)

In this study, 4 experimental groups of bovine blastocysts were generated, namely (I) VITRO (fully in vitro derived embryos), (II) IFOT-M (intrafollicular transfer of in vitro matured oocytes into preovulatory follicles) and (III) IFOT-IM (intrafollicular transfer of immature oocytes into dominant follicles), and (IV) fully in vivo derived embryos as control (VIVO). For the VITRO embryos, a total of 866 immature oocytes collected from the abattoir in 13 replicates achieved a mean cleavage rate of 83.7 ± 5.3% after in vitro fertilization. Of these, 34.8 ± 9.5% reached the morula or blastocyst stage after in vitro culture up to day 7 (Table 1). Considering IFOT-M embryos, a total of 1646 abattoir-derived oocytes were transferred into the preovulatory follicles of 28 synchronized recipients after in vitro maturation. Of these, 36.8 ± 29.4% were retrieved by uterine flushing 7 days later. These showed a cleavage rate of 69.2 ± 21.0% and, a development rate to morula or blastocyst of 48.5 ± 26.2% based on cleaved embryos (Table 1). For IFOT-IM embryos, a total of 791 abattoir-derived immature oocytes were transferred into the dominant follicle of 16 synchronized recipients immediately after ovarian retrieval. Of these, 38.5 ± 27.4% were retrieved by uterine flushing 7 days later. These showed a cleavage rate of 83.2 ± 35.1% and, a development rate to morula or blastocyst of 48.8 ± 25.6% from the cleaved ones (Table 1). Finally, when 8 donor recipients were flushed for collection of VIVO embryos, a total of 115 cleavage stage embryos were recovered. These showed a cleavage rate of 74.2 ± 12.2% and, a development rate to morula or blastocyst of 85.1 ± 11.7% (out of the cleaved zygotes). While embryo recovery rates did not differ between IFOT-M and IFOT-IM groups, cleavage rates were also comparable between experimental groups. IFOT-M and IFOT-IM embryos did not show higher developmental rates to the morula or blastocyst stage compared to VITRO embryos, while VIVO embryos significantly outperformed all other groups in terms of developmental rate (*p* < 0.05, Table 1). Finally, when 8 donor-recipients were flushed for VIVO embryo collection, a total of 115 different stages of embryos were obtained. These showed a cleavage rate of 74.2 ± 12.2% and, a development rate to morula or blastocyst of 85.1 ± 11.7% (out of the total cleaved zygotes). When comparing the morphology of these embryos under the light microscope, VITRO embryos appeared darker and opaquer compared to IFOT-M and IFOT-IM embryos (Fig. 2, A-C). This finding was further supported by comparing individual embryos at higher magnification (Fig. 2, D-G).

**Table 1:** Developmental metrics of embryos developed in contrasting environments

	Replicates	Total	(Re)collected		Cleaved		Mo	BL	Mo+BL	MoBL/Total	MoBL/Cleaved
Treatment	(n)	(n)	(n)	(Mean ± SD)	(n)	(Mean ± SD)	(n)	(n)	(n)	(Mean ± SD)	(Mean ± SD)
VITRO	13	866			726	83.7 ± 5.3 %	123	125	248	29.3 ± 8.6 % ^a^	34.8 ± 9.5 % ^a^
IFOT-M	28	1646	610	36.8 ± 29.4 %	414	69.2 ± 21.0 %	89	77	166	32.8 ± 20.1 % ^a^	48.5 ± 26.2 % ^a^
IFOT-IM	16	791	306	38.5 ± 27.4 %	250	83.2 ± 35.1 %	85	62	147	40.1 ± 22.2 % ^a^	48.8 ± 25.6 % ^a^
VIVO	8		115		89	74.2 ± 12.2 %	33	43	76	63.5 ± 14.8 % ^b^	85.1 ± 11.7 % ^b^

*Mo *Morula, *BL *Blastocyst, *MoBL *Morula or Blastocyst, different superscript indicate significantly different values (*p*<0.05)

### Differential expressed genes (DEG) among contrasting experimental groups

**Fig. 3:**
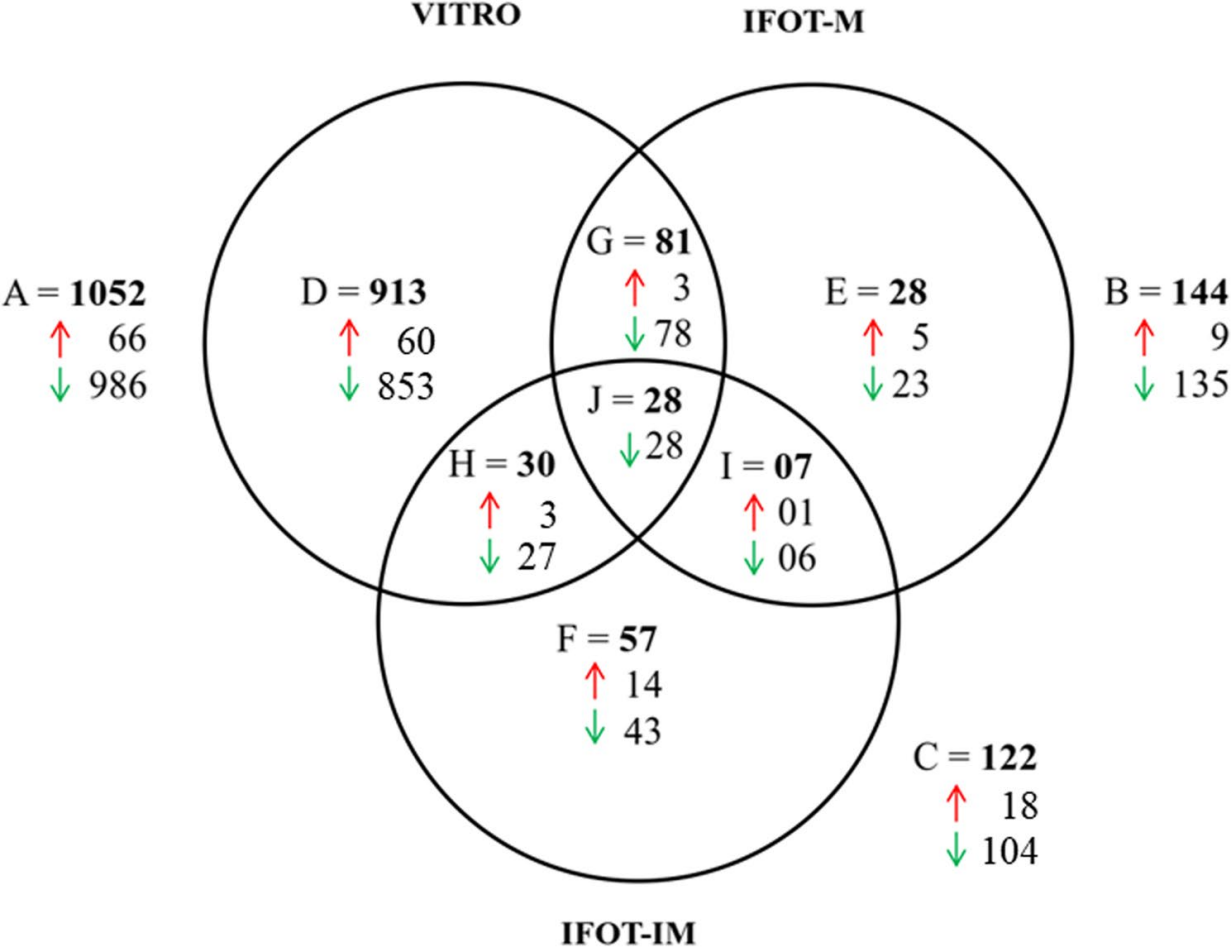
Venn diagram demonstrating the number of differentially up and down-regulated genes (DEGs) in VITRO, IFOT-M and IFOT-IM embryos (FC > 2, FDR < 0.1) relative to VIVO embryos. (**A**, VITRO) DEGs of entirely in vitro developed blastocysts in comparison to VIVO embryos. (**B**, IFOT-M) DEGs of embryos matured in vitro and transferred into a preovulatory follicle to allow in vivo development up to the blastocyst stage in comparison to VIVO embryos. (**C**, IFOT_IM) DEGs of embryos derived from slaughterhouse ovaries and transferred into a dominant follicle to allow in vivo maturation and development up to the blastocyst stage in comparison to VIVO embryos. **D** Genes differentially regulated specifically because of the environment after maturation. **G**&**E** Genes differentially regulated specifically because of the environment during maturation. **J** Highly confirmed genes differentially expressed due to oocyte source. Up- and down-regulated genes are presented by red and green arrows, respectively

Hierarchical clustering was performed to figure out the global gene expression outline in groups of embryos produced in contrasting experimental conditions (VIVO, VITRO, IFOT-M, IFOT-IM). As a result, the present study identified four hierarchical heatmap cluster representing the gene expression level of VIVO, VITRO, IFOT-M and IFOT-IM embryos, respectively (Supplemental Fig. 3). Comparison of these gene expression level between embryo groups allowed investigation of differentially expressed genes in 3 contrasting embryo groups developed in contrasting developmental environments (VITRO, IFOT-M, IFOT-IM) in comparison with VIVO embryos. Irrespective of chosen false discovery rate (FDR) VITRO embryos showed a much higher number of DEGs (Fold change > 2) compared to VIVO embryos than IFOT-M or IFOT-IM embryos (Table 2). For further downstream analysis DEGs (FC > 2, raw *p*-value < 0.05, False discovery rate (FDR) < 0.1) were selected. Only a minority of DEGs was up-regulated in VITRO, IFOT-M and IFOT-IM embryo groups compared to VIVO embryos. For example, only 66 out of 1052 DEGs were up-regulated in VITRO embryos. Similarly, only 9 (out of 144) and 18 (out of 122) DEGs were up-regulated in embryos derived from IFOT-M and IFOT-IM, respectively. The DEGs unique to VITRO, IFOT-M or IFOT-IM embryos followed the same trend, too. Only 60 (out of 913), 5 (out of 28), and 14 (out of 57) DEGs were up-regulated in VITRO, IFOT-M, and IFOT-IM embryos compared to VIVO embryos, respectively. An overview of commonly and exclusively DEGs among the three experimental embryo groups relative to VIVO embryos is presented in Fig. 3.

**Table 2:** Number of differential expressed genes among groups depending on choice of False discovery rate

		**False discovery rates**			
		**none**	**(FDR < 0.10)**	**(FDR < 0.05)**	**(FDR < 0.01)**
VITRO vs. VIVO	Up	77	66	38	4
	Down	1000	986	848	162
	**Total**	**1077**	**1052**	**886**	**166**
IFOT-M vs. VIVO	Up	17	9	4	0
	Down	155	135	95	0
	**Total**	**172**	**144**	**99**	**0**
IFOT-IM vs. VIVO	Up	24	18	6	0
	Down	132	104	25	0
	**Total**	**156**	**122**	**31**	**0**

Number of differentially expressed up- or down-regulated genes in different groups (Fold change >2) with different FDRs

### System effect of in vitro embryo production on blastocyst transcriptome outline

**Fig. 4:**
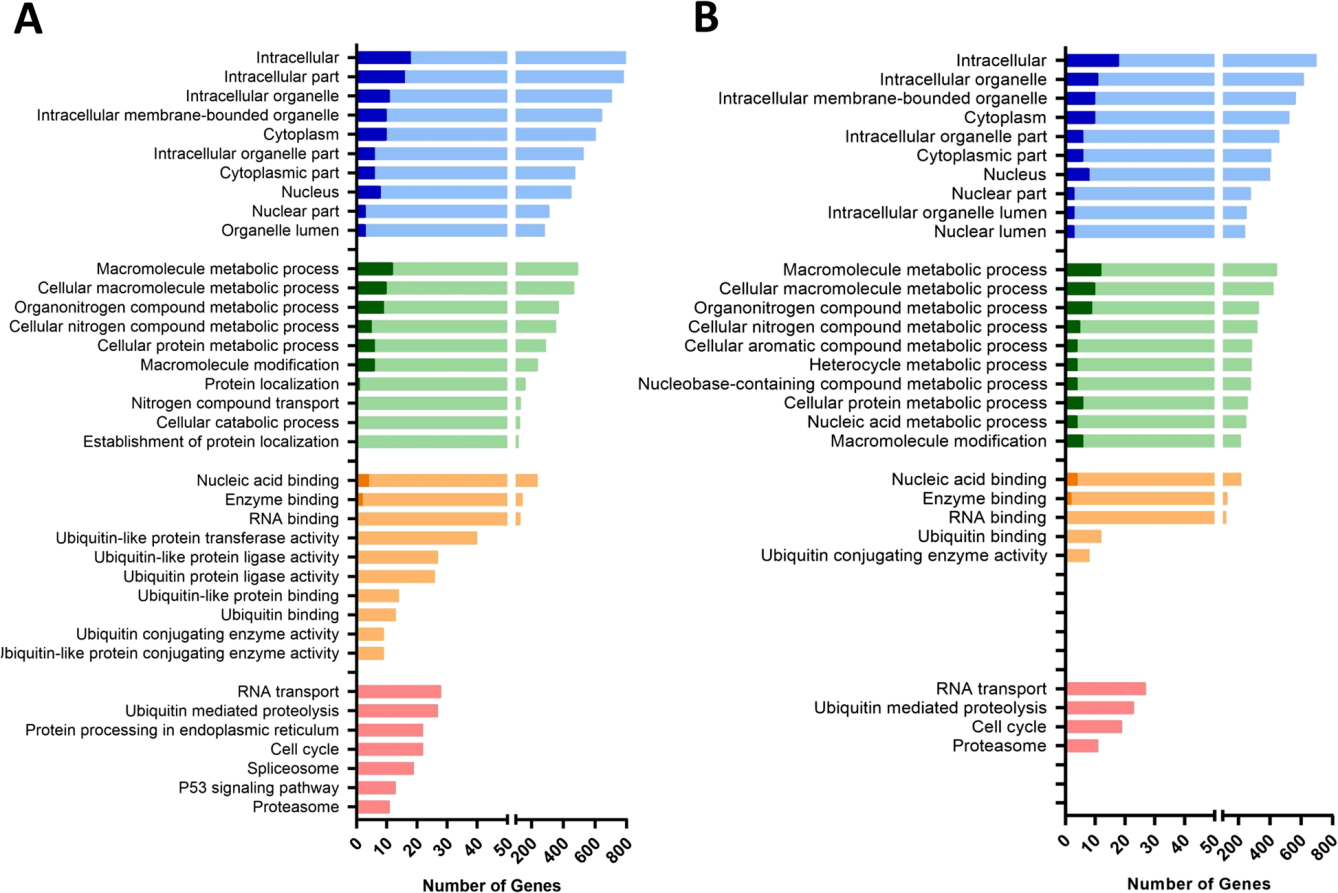
**A** Top Cellular components (blue), Biological processes (green), Molecular functions (orange) and KEGG pathways (red) enriched in genes differentially expressed between VITRO and VIVO embryos (FC > 2, FDR < 0.1). **B** Top Cellular components (blue), Biological processes (green), Molecular functions (orange) and KEGG pathways (red) enriched in genes differentially expressed between IFOT-M and VIVO embryos (FC > 2, FDR < 0.1). Solid colors represent up-regulated genes and light colors represent down-regulated genes

**Fig. 5:**
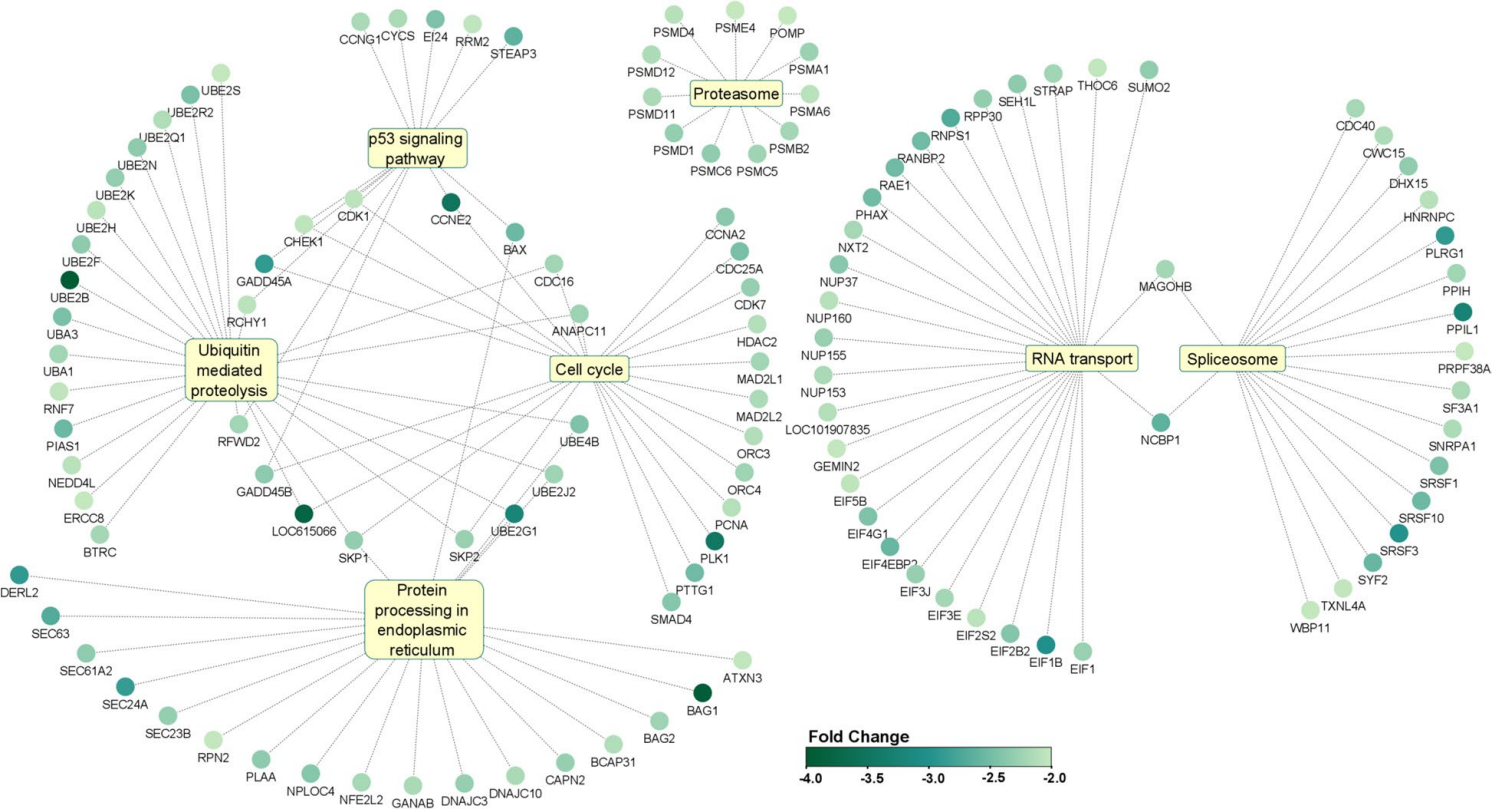
Functional annotation and sub-network enrichment analysis identified 7 KEGG pathways sharing differentially expressed genes in VITRO blastocysts. Of these, 5 KEGG pathways including “RNA transport”, “Ubiquitin mediated proteolysis”, “Protein processing in endoplasmic reticulum”, “Spliceosome” and “Proteasome” play a role for protein synthesis or degradation. In addition, 2 KEGG pathways namely “cell cycle” and “p53 signalling pathway” play a fundamental role for cell cycle regulation of the early embryo. The color intensity of differentially regulated genes among these pathways correlates with their level of down-regulation (light to solid color)

In total, 1052 DEGs were identified between fully in vitro derived bovine embryos (VITRO) compared to in vivo derived embryos (VIVO, Additional File 2). The vast majority of these transcripts were down-regulated (986/1052, 94%) whereas only 66 out of 1052 (6%) were up-regulated in IVP embryos. Further gene ontology analysis revealed that these DEGs play a significant role in 61 cellular components with “intracellular” the most affected one (796 DEGs, 97.7% down-regulated) followed by “intracellular part” (782 DEGs, 97.8% down-regulated), “intracellular organelle” (707 DEGs, 98% down-regulated), “intracellular membrane-bounded organelle” (645 DEGs, 98.5% down-regulated) as well as “cytoplasm” (605 DEGs, 98.4% as presented in Fig. 4A). In terms of involvement in biological pathways our study revealed 33 biological processes to be significantly affected by DEGs between VITRO and VIVO embryos. Of these “macromolecule metabolic process” was the most affected pathway (494 DEG) with 97.6% of them being down-regulated due to IVP followed by “cellular macromolecule metabolic process” (469, 97.9% down-regulated), “organonitrogen compound metabolic process (372, 97.6% down-regulated), “cellular nitrogen metabolic process” (354, 98.6% down-regulated) and “cellular protein metabolic process” (290, 97.9% down-regulated) as shown in Fig. 4A. In addition, these DEGs were identified to play a significant role in 10 molecular functions with “nucleic acid binding” being the most affected one (237 DEGs, 98.3% down-regulated) followed by “enzyme binding” (143 DEGs, 98.6% down-regulated), “RNA binding” (129 DEGs, 100% down-regulated), “ubiquitin-like protein transferase activity” (40 DEGs, 100% down-regulated) as well as “ubiquitin-like protein ligase activity” (27 DEGs, 100% down-regulated) as demonstrated in Fig. 4A. Ultimately, further analysis identified 7 KEGG pathways affected by these DEGs between VITRO and VIVO embryos (Additional File 2). Of these, the 5 KEGG pathways “RNA transport”, “Ubiquitin mediated proteolysis”, “Protein processing in endoplasmic reticulum”, “Spliceosome” as well as “Proteasome” play a role in Protein synthesis and/or degradation. In detail, “RNA transport” contained among others 9 DEGs of the Eukaryotic Translation Initiation Factor (EIF) family and 4 DEGs (NUP37, NUP160, NUP155, NUP153) of the Nucleoporin (NUP) -family. “Ubiquitin mediated proteolysis” encompassed 11 DEGs of the Ubiquitin Conjugating Enzyme (UBE)– family. “Spliceosome” encompassed 3 DEGs (SRSF1, SRSF10, SRSF3) of the Serine and Arginine Rich Splicing Factor (SRSF)– family and “Proteasome” contained 11 DEGS being part of the Proteasome 20S Subunit Alpha (PSMA1, PSMA6), Proteasome 20S Subunit Beta (PSMB2), Proteasome 26S Subunit (PSMC5, PSMC6, PSMD1, PSMD4, PSMD11, PSMD12), Proteasome Activator Subunit (PSME4)-families (Fig. 5). In agreement, 2 KEGG pathways namely “cell cycle” containing among others Checkpoint Kinase 1 (CHEK1), Cyclin Dependent Kinase 1 (CDK1), Cyclin E2 (CCNE2), Cyclin A2 (CCNA2), Cell Division Cycle 25 A (CDC25A), Serine/threonine-protein kinase 1 (PLK1) as well as Anaphase Promoting Complex Subunit 11 (ANAPC11) and “p53 signalling pathway” encompassing again CHEK1, CDK1, CCNE2 but also Apoptosis Regulator BAX (BAX) play a fundamental role for cell cycle regulation of the early embryo (Fig. 5).

### Sustained impact of the post-maturation environment on blastocyst transcriptome profile

**Fig. 6:**
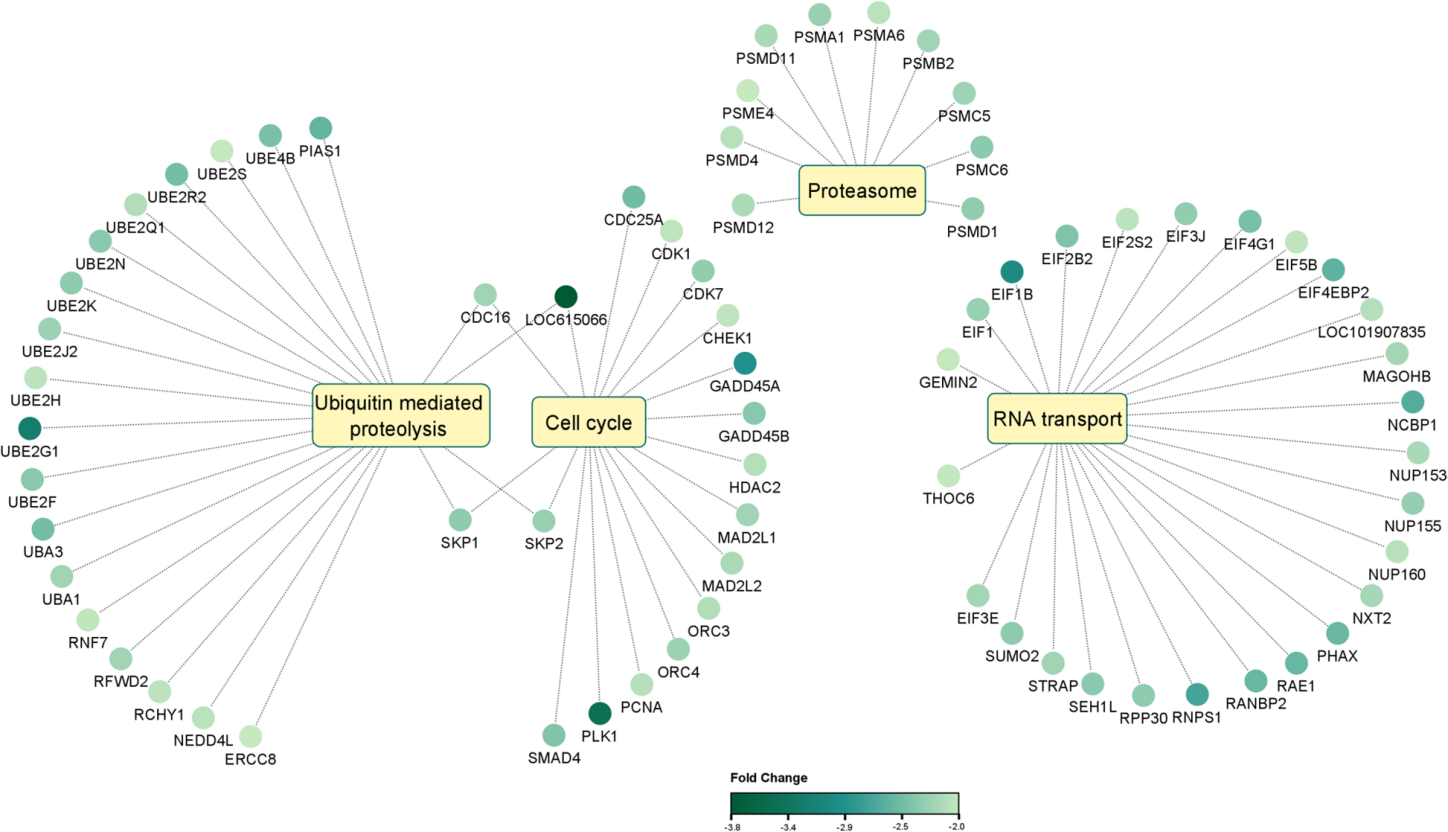
Functional annotation and sub-network enrichment analysis identified 4 KEGG pathways sharing differentially expressed genes in IFOT-M blastocysts. Of these, 3 KEGG pathways including “RNA transport”, “Ubiquitin mediated proteolysis”, and “Proteasome” play a role for protein synthesis or degradation. In addition, 1 KEGG pathway namely “cell cycle” plays a fundamental role for cell cycle regulation of the early embryo. The color intensity of differentially regulated genes among these pathways correlates with their level of down-regulation (light to solid color)

The great majority of DEGs of group VITRO (913 of 1052, 86.8%) was not differentially expressed in IFOT-M or IFOT-IM embryos, too. To investigate the exclusive effect of the environment after in vitro maturation, DEGs common between VITRO and IFOT-M or IFOT-IM embryos were therefore excluded from further gene ontology analysis (Fig. 3, Venn D). The vast majority of these transcripts (853 of 913, 93.4%) was down-regulated compared to VIVO embryos (Additional File 5). Gene ontology analysis of these DEGs (*n* = 913) confirmed that these play a role in 42 cellular components (Fig. 4B) with “intracellular” found to be the most affected one (699 DEGs, 97.4% down-regulated) followed by “intracellular part” (686 DEGs, 97.7% down-regulated), “intracellular organelle” (618 DEGs, 98.2% down-regulated), “intracellular membrane-bounded organelle” (567 DEGs, 98.2% down-regulated) as well as “cytoplasm” (524 DEGs, 98.1% down-regulated) as presented in Fig. 4B. Biological pathway analysis identified a total of 27 biological processes, including “macromolecule metabolic process” (444 DEGs, 97.3% down-regulated), “cellular macromolecular metabolic process” (424 DEGs, 97.6% down-regulated), “organonitrogen compound metabolic process” (331 DEGs, 97.3% down-regulated), “cellular nitrogen compound metabolic process” (320 DEGs, 98.4% down-regulated) and “cellular aromatic compound metabolic process” (286 DEGs, 98.6% down-regulated) as presented in Fig. 4B. Furthermore, DEGs play a significant role in 6 molecular functions (Fig. 4B) with “nucleic acid binding” found to be the most affected one (218 DEGs, 98.2% down-regulated) followed by “enzyme binding” (129 DEGs, 98.45% down-regulated), “RNA binding” (121 DEGs, 100% down-regulated), “ubiquitin binding” (12 DEGs, 100% down-regulated) as well as “ubiquitin conjugating enzyme activity” (8 DEGs, 100% down-regulated) as demonstrated in Fig. 4B. Further analysis identified 4 KEGG pathways (Additional File 5) namely “RNA transport”, “Ubiquitin mediated proteolysis”, “Cell cycle” and “Proteasome” were identified to be fundamentally affected by these genes exclusively differentially expressed between VITRO and VIVO embryos (Fig. 4B). Of these, the 3 KEGG pathways “RNA transport”, “Ubiquitin mediated proteolysis”, as well as “Proteasome” play a role in Protein synthesis and/or degradation. In detail, “RNA transport” contained among others 9 DEGs of the Eukaryotic Translation Initiation Factor (EIF) family and 4 DEGs (NUP37, NUP160, NUP155, NUP153) of the Nucleoporin (NUP) -family. “Ubiquitin mediated proteolysis” encompassed 10 DEGs of the Ubiquitin Conjugating Enzyme (UBE)– family with Ubiquitin Conjugating Enzyme E2 (UBE2G1) being most down-regulated. Finally, the “Proteasome” pathway contained 10 DEGS being part of the Proteasome 20 S Subunit Alpha (PSMA1, PSMA6), Proteasome 20 S Subunit Beta (PSMB2), Proteasome 26 S Subunit (PSMC5, PSMC6, PSMD1, PSMD4, PSMD11, PSMD12) and Proteasome Activator Subunit (PSME4)-families (Fig. 6). Moreover, 1 KEGG pathways namely “cell cycle” containing among others Checkpoint Kinase 1 (CHEK1), Cyclin Dependent Kinase 1 (CDK1), Cell Division Cycle 25 A (CDC25A) as well as Serine/threonine-protein kinase 1 (PLK1) plays a fundamental role for cell cycle regulation of the early embryo (Fig. 6).

### Sustained impact of the maturation environment on the blastocyst transcriptome profile

**Fig. 7:**
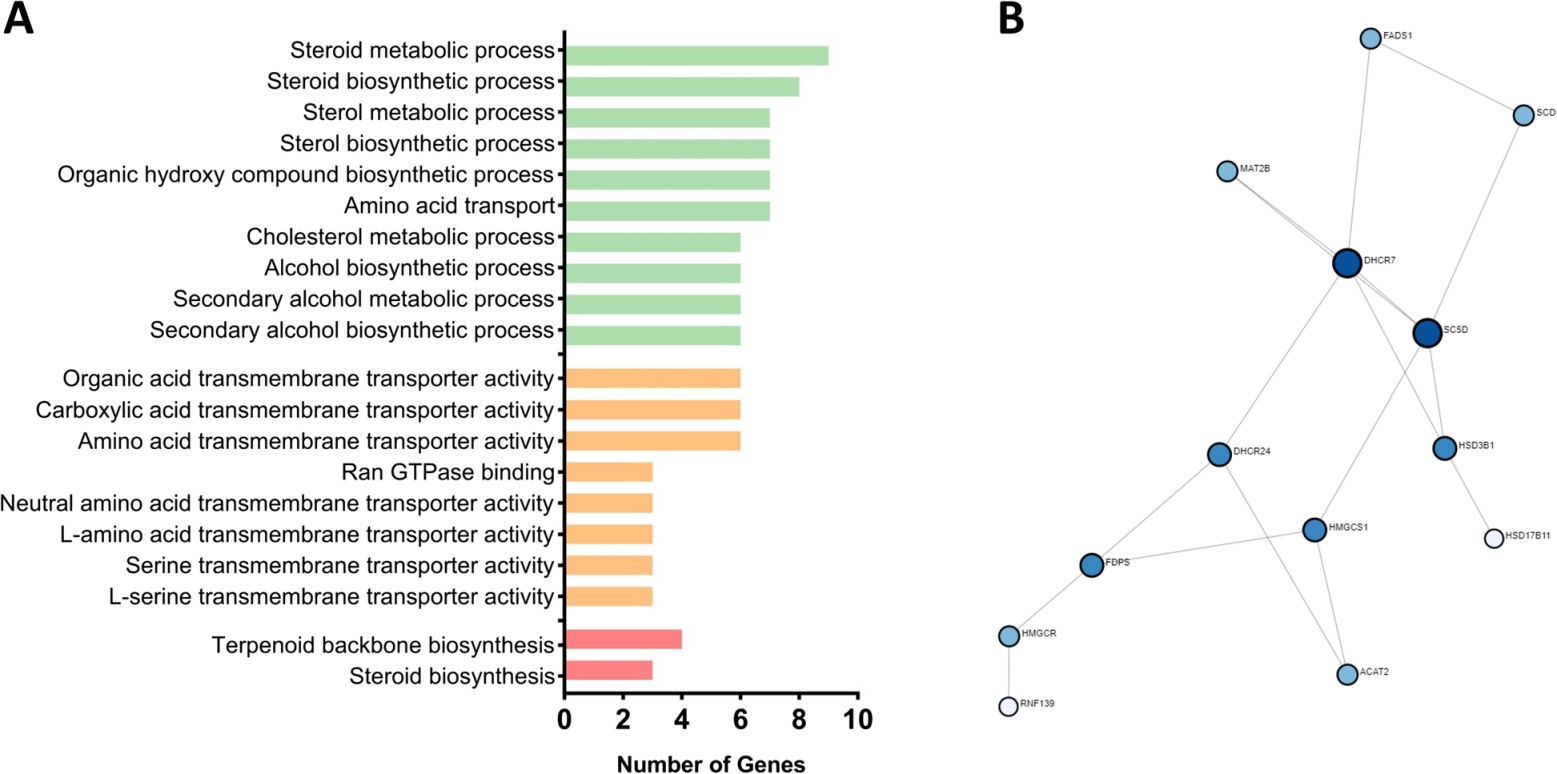
**A** Top Biological processes (green), Molecular functions (orange) and KEGG pathways (red) enriched in genes differentially expressed between IFOT-IM and VIVO embryos (FC > 2, FDR < 0.1). Solid colors represent up-regulated genes and light colors represent down-regulated genes. **B** Among these differentially expressed genes, analysis of Protein-Protein Interactions (zero order network, confidence score = 900) predicted 13 highly interconnected genes with DHCR7, DHCR24, SC5D, HMGCR and HMGCS1 playing an outstanding role specifically due to the in vitro environment after maturation

Comparison of group IFOT-M with group VIVO embryos (Fig. 3, Venn B) identified a total of 144 DEGs with 135 (94%) of them down-regulated relative to VIVO embryos (Additional File 3). A total of 109 genes were not differentially expressed in IFOT-IM embryos, too. To investigate the exclusive effect of the environment during in vitro maturation, DEGs common between IFOT-M and IFOT-IM embryos were therefore excluded from further gene analysis (Additional File 6). Gene ontology analysis found that DEGs affected by the environment during maturation play a significant role in 21 biological processes with “steroid metabolic process” (9 DEGs) representing the most affected pathway followed by “steroid biosynthetic process” (8 DEGs), “organic hydroxyl compound biosynthetic process” (7 DEGs), “sterol metabolic process” (7 DEGs), “sterol biosynthetic process” (7 DEGs) as well as “amino acid transport” (7 DEGs) as demonstrated in Fig. 7A. In addition, biological pathway analysis revealed that these DEGs play a role in 8 molecular functions. Of these, “organic acid transmembrane transporter activity” (6 DEGs), “carboxylic acid transmembrane transporter activity” (6 DEGs), “amino acid transmembrane transporter activity” (6 DEGs) were identified to be most affected (Fig. 7A). Finally, pathway analysis observed 2 KEGG pathways affected by the environment during maturation namely “Terpenoid backbone synthesis” encompassing 4 DEGs (ACAT2, FDPS, HMGCR, HMGCS1) as well as “Steroid biosynthesis” encompassing 3 DEGs (DHCR24, DHCR7, SC5D) as demonstrated in Fig. 7A. Noteworthy, all DEGs within these two pathways were down-regulated in comparison to VIVO embryos. Protein-Protein Interactome (PPI) analysis (confidence score = 900, zero order network) predicted 13 highly interconnected genes with HMGCR, HMGCS1, DHCR24, DHCR7, SC5D playing an outstanding role among these 109 DEGs differential expressed in IFOT-M but not in IFOT-IM embryos (Fig. 7B).

### Sustained impact of the oocyte source on the blastocyst transcriptome landscape

**Fig. 8:**
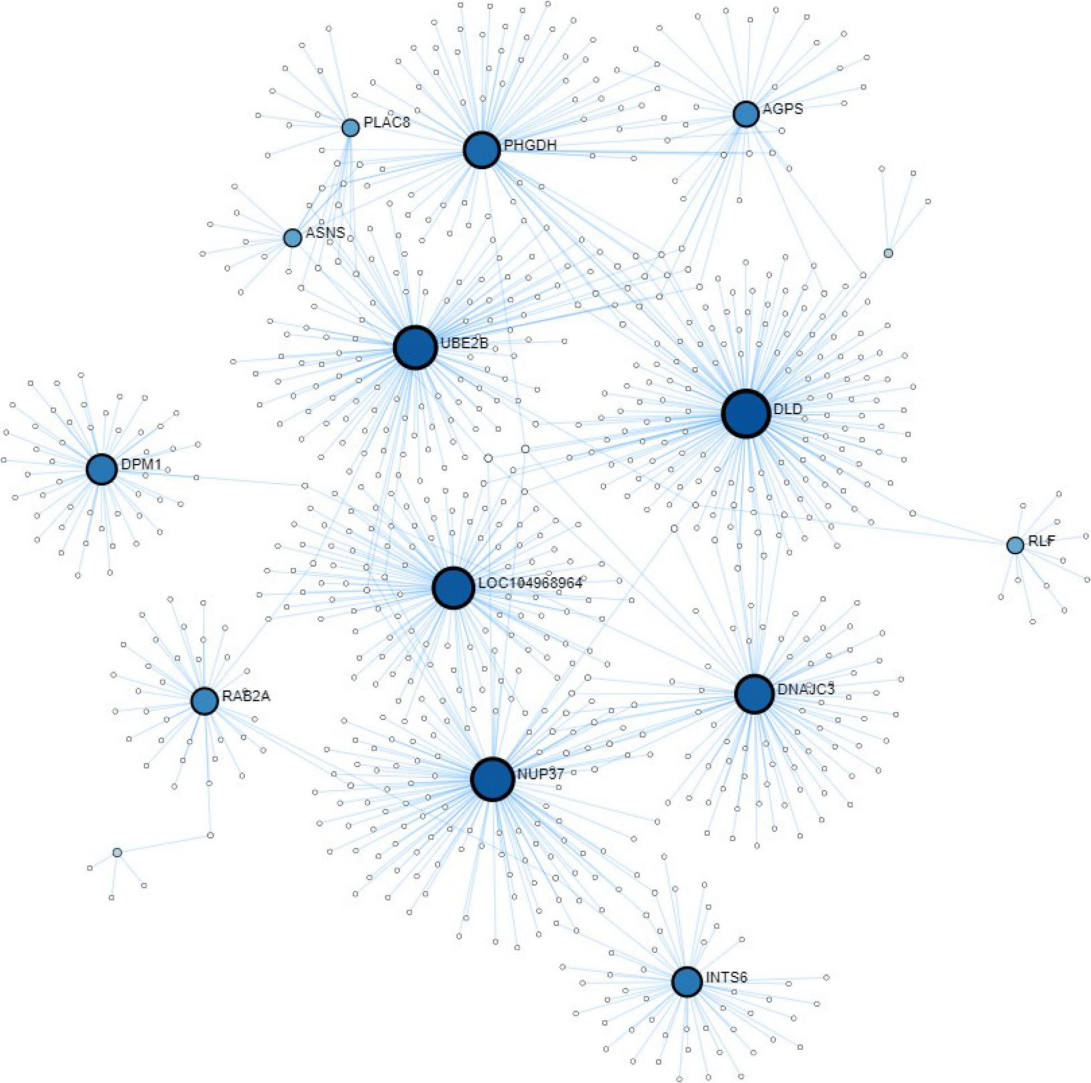
Analysis of Protein-Protein Interactions (second order network, confidence score = 401) predicted 13 highly interconnected genes with DLD, ACTC1, UBE2B, DNAjC3, Rab2A as well as PLAC8 playing an outstanding role among the 28 commonly differentially expressed genes in VITRO, IFOT-M and IFOT-IM derived blastocysts

Oocytes collected from slaughterhouse ovaries and transferred immediately into a dominant follicle (IFOT-IM) allowing in vivo maturation, in vivo fertilization as well as in vivo early embryo development were compared against in vivo derived blastocyst (VIVO). IFOT-IM showed 122 DEGs compared to VIVO embryos (Fig. 3). When comparing IFOT-IM embryos with VIVO embryos (VIVO) one biological process namely “modification of host by symbiont morphology or physiology” were found to be affected. Likewise, one KEGG pathway, namely “steroid biosynthesis” encompassing Cytochrome P450 Family 51 Subfamily A Member 1 (CYP51A1), Methylsterol Monooxygenase 1 (MSMO1) and Squalene Epoxidase (SQLE) was obtained to be affected by oocyte source (Additional File 4). Among these DEGs a total of 28 genes was shared between VITRO, IFOT-M and IFOT-IM in relation to VIVO embryos, respectively (Fig. 3). Therefore, these DEGs represent higher confirmed candidates affected by the source of ovaries. Remarkably, all 28 DEGs were found to be down-regulated in VITRO, IFOT-M and IFOT-IM compared to VIVO embryos (Table 3, Additional File 7). Protein-Protein Interactome (PPI) analysis (confidence score 401, second order network) predicted 13 highly interconnected genes with DLD, ACTC1, UBE2B, DNAjC3, Rab2A as well as PLAC8 playing an outstanding role among these 28 DEGs genes differentially expressed in common in VITRO, IFOT-M and IFOT-IM derived blastocysts (Fig. 8).

**Table 3:** Differentially expressed genes shared between embryos of VITRO, IFOT-M and IFOT-IM groups

Gene	Ensembl Transcript IDs	Description	Chr	FC	FDR
ACTC1	ENSBTAT00000007504	actin, alpha, cardiac muscle 1	chr10	-4.76	0.0182
GALNT1		polypeptide N-acetylgalactosaminyltransferase 1	chr24	-4.62	0.0088
TMEM165	ENSBTAT00000001671	transmembrane protein 165	chr6	-4.24	0.0068
UBE2B		ubiquitin-conjugating enzyme E2B	chr7	-3.95	0.0118
ASNS	ENSBTAT00000004181	asparagine synthetase (glutamine-hydrolyzing)	chr4	-3.49	0.0012
LOC104968964	ENSBTAT00000065846	guanine nucleotide-binding protein G(I)/G(S)/G(O) subunit gamma-5	chr7	-3.36	0.0459
CD59	ENSBTAT00000002967	CD59 molecule, complement regulatory protein	chr15	-3.35	0.0134
NID2	ENSBTAT00000029257	nidogen 2 (osteonidogen)	chr10	-3.18	0.0358
INTS6		integrator complex subunit 6	chr12	-3.10	0.0068
RAB2A	ENSBTAT00000001253	RAB2A, member RAS oncogene family	chr14	-2.99	0.0266
DLD	ENSBTAT00000033787	dihydrolipoamide dehydrogenase	chr4	-2.98	0.0061
GARS	ENSBTAT00000025254	glycyl-tRNA synthetase; glycine--tRNA ligase-like	chr4	-2.81	0.0048
PHGDH	ENSBTAT00000008907	phosphoglycerate dehydrogenase	chr3	-2.79	0.0255
SRI	ENSBTAT00000053811	sorcin	chr4	-2.79	0.0174
SLC35G1	ENSBTAT00000048596	solute carrier family 35, member G1	chr26	-2.74	0.0144
SLC38A9	ENSBTAT00000047351	solute carrier family 38, member 9	chr20	-2.69	0.0068
AGPS		alkylglycerone phosphate synthase	chr2	-2.63	0.0273
PTPLB	ENSBTAT00000032276	protein tyrosine phosphatase-like (proline instead of catalytic arginine), member b	chr1	-2.50	0.0088
SPIC	ENSBTAT00000013069	Spi-C transcription factor (Spi-1/PU.1 related)	chr5	-2.47	0.0147
RLF	ENSBTAT00000026252	rearranged L-myc fusion	chr3	-2.43	0.0163
NUP37	ENSBTAT00000000024	nucleoporin 37kDa	chr5	-2.38	0.0188
ULBP17	ENSBTAT00000057496	UL16-binding protein 17; retinoic acid early transcript 1G	chr9	-2.38	0.0110
RAET1G	ENSBTAT00000057496	UL16-binding protein 17; retinoic acid early transcript 1G	chr9	-2.38	0.0110
UNC50	ENSBTAT00000019807	unc-50 homolog (C. elegans)	chr11	-2.37	0.0109
DPM1	ENSBTAT00000010153	dolichyl-phosphate mannosyltransferase polypeptide 1, catalytic subunit	chr13	-2.33	0.0138
DNAJC3	ENSBTAT00000016001	DnaJ (Hsp40) homolog, subfamily C, member 3	chr12	-2.29	0.0111
MCOLN2	ENSBTAT00000066065	mucolipin 2	chr3	-2.15	0.0154
PLAC8	ENSBTAT00000012987	placenta-specific 8	chr6	-2.13	0.0061

*Chr *Chromosome, *FC *Fold change, *FDR *False discovery rate

## Discussion

Although the four contrasting groups of embryos did not differ in cleavage rates, VIVO embryos exhibited significantly higher rates of advanced development, reaching the morula and/or blastocyst stage by Day 7, both in terms of total number and relative percentage to the number of cleaved embryos. In other words, the source of immature oocytes influenced the maximumal developmental capacity of the resulting embryos. These findings are consistent with our previous reports on intrafollicularly transferred oocytes [21, 22] and corroborate earlier studies by others [2, 20]. Moreover, VITRO embryos appeared opaquer than those from all other groups that underwent early development within the oviduct. Given that opacity is associated with increased lipid content, these observations, previously reported by our group [21], align with earlier studies showing that the post-maturation environment primarily influences embryo quality, whereas the oocyte origin and maturation environment predominantly affect the rate of blastocysts formation [2, 20].

The present study identified four major hierarchical cluster separating VIVO embryos from IFOT-IM, IFOT-M and VITRO embryos, respectively. These results are in line with earlier studies demonstrating that the environment after fertilization determines the embryo quality at the blastocyst stage [2, 20]. All in all, our study observed a dramatically higher number of DEGs in fully in vitro produced embryos (VITRO) compared to those that had been transferred after in vitro maturation to the physiological environment of the preovulatory follicle (IFOT-M) allowing fertilization and further development in vivo. Similarly, embryos of the VITRO group showed a significantly higher number of DEG compared to those transferred at immature stage into the dominant follicle (IFOT-IM) enabling in vivo maturation, fertilization and development to the blastocyst stage both in comparison with VIVO embryos serving as “traditionally accepted gold standard”, respectively. These findings are in line with one of our recent studies indicating higher numbers of DEG the earlier embryos are flushed from the physiological environment [4]. This might be explained by the effect of the non-physiological environment at time of embryonic genome activation (EGA) in VITRO embryos whereas IFOT-IM and IFOT-M groups experienced EGA within the oviduct representing the physiological environment. Indeed, the environment at time of EGA has been shown to exhibit great effects on the gene expression profile of bovine embryos [4].

### Systemic effects of in vitro embryo production on blastocyst transcriptome fingerprint

The present study clearly demonstrated marked differences in terms of the global gene expression outline of IVP derived bovine embryos compared to in vivo derived embryos. The vast majority of these DEGs was found to be down-regulated in comparison with in vivo derived embryos as reported earlier [4]. These DEGs played above all a role within intracellular components, especially within the cytoplasm. With respect to biological processes, especially macromolecule metabolic processes as well as protein metabolic processes were affected. With respect to biological functions especially those involved in RNA binding and ubiquitin-like protein activity were affected. Of impact, our study revealed that IVP derived embryos distinguish from in vivo derived embryos predominately in three main pathway outlines, namely protein synthesis, protein degradation and cell-cycle regulation. These findings are in great accordance to the general accepted finding that in vitro culture conditions can impact gene expression signatures [1, 2] affecting the quality of embryos produced through assisted reproductive techniques [20]. Maternal mRNAs deposited in the oocyte are in turn critical for the initial stages of development, including the regulation of zygotic genome activation (ZGA) [30] representing a pivotal event in early embryogenesis [31, 32]. Moreover, the observed environmental effects of the present study are in agreement with earlier findings indicating that environmental factors such as pH, temperature and osmolarity in the culture medium might influence embryonic RNA synthesis [20, 33, 34] as well as DNA methylation and histone modifications, which in turn can affect the transcriptional activation of key genes during ZGA [35]. In contrast, our study showed that genes differentially expressed between in vitro and in vivo derived blastocysts play fundamental roles in pathways like “Spliceosome”, “protein processing in endoplasmic reticulum” and “P53 pathway”.

With respect to the “Spliceosome” pathway, embryos undergo significant transcriptional and epigenetic changes and the regulation of gene expression, including splicing, is tightly controlled to ensure the accurate execution of developmental programs [36]. Disruptions in splicing can interfere with essential developmental processes, leading to defects or developmental arrest [37]. In great accordance to our results, the composition of the culture medium, including its ionic balance, nutrients, and pH as well as plastic ware has been reported to affect splicing [38–40]. In addition, the present study identified dysregulation of “Protein processing in endoplasmic reticulum” to be a unique characteristic of bovine IVP derived embryos. Since proper folding is crucial for functional proteins, the endoplasmic reticulum (ER), which plays a critical role in protein synthesis, folding and post-translational modifications, is thought to be essential for the proper functioning of the cell. Thus, we speculate that quality and capacity of “Protein processing in endoplasmic reticulum” affects viability and developmental capacity of bovine embryos and dysregulation of “protein processing in endoplasmic reticulum” in in vitro derived embryos may provide a clue for that. Indeed, ER stress has been shown to compromise the development and cryo-tolerance in bovine [41] and porcine [42] embryos. Vice versa, ER stress reducing agents have been demonstrated to increase quality of oocytes and embryos [43, 44] as well as implantation rates after transfer of murine embryos to recipients [45]. The ER in turn has been shown to be particularly sensitive to oxidative stress [46, 47] resulting in impaired protein folding and increased apoptosis [48]. Finally, the present study identified dysregulation of the “P53 pathway” to be a unique characteristic of bovine IVP derived embryos. The p53 protein is referred to as the “guardian of the genome” and primarily functions as a transcription factor that can induce cell cycle arrest, apoptosis, or senescence [49]. While p53 upregulates the expression of p21, a cyclin-dependent kinase inhibitor that holds the cell cycle at the G1/S checkpoint, allowing for DNA repair [50]. Therefore, we suggest that the p53 pathway is particularly important in preimplantation embryos, due to their rapid cell divisions and significant epigenetic reprogramming occurring during this stage with only proper functioning of p53 ensuring that embryos can respond appropriately to DNA damage, thus maintaining genomic stability and viability. In agreement to the results of our study, it has been demonstrated that environmental factors such as temperature [51] and oxygen levels [52] bear potential to alter p53 activity.

### The sustained impact of the post-maturation environment on blastocyst transcriptome profile

To investigate the specific effect of the environment after in vitro maturation, only DEGs between VITRO and VIVO but not between IFOT-M and VIVO were considered. It became obvious, that the great majority of DEGs in embryos of group VITRO was not differentially expressed in IFOT-M, too. Thus, the vast majority of these DEGs were particularly influenced by the post-maturation environment, which under physiological conditions corresponds to the oviduct and the uterine horns. Further analysis revealed that this post-maturation environment significantly impacted the expression of genes involved in fundamental cellular processes, including protein synthesis, protein degradation and cell-cycle regulation. Among protein synthesis related pathways, our study identified that differential regulated genes affected by the in vitro environment after maturation are involved in the “RNA transport” pathway. In early embryos, RNA transport mechanisms are ensur the localization of mRNAs to specific cellular regions, a process that is critical for establishing cell polarity, axis formation and proper tissue differentiation [53]. Mislocalization of mRNAs on the other hand, can result in aberrant protein expression, leading to developmental defects or developmental arrest [54]. For instance, disruptions in the localization of mRNAs involved in cell cycle regulation and apoptosis have been shown to impair blastocyst formation and reduce implantation success rates [32]. Optimizing the in vitro culture conditions to better support efficient RNA transport may therefore hold significant potential for improving embryo quality and enhancing the success rates of ART, including in vitro fertilization (IVF) and embryo transfer [55]. Among the pathways playing a role in protein degradation, the present study revealed that genes differentially expressed particularly due to the environment after maturation play fundamental roles in “ubiquitin mediated proteolysis” as well as “Proteasome”-related pathways. Ubiquitin-mediated proteolysis tags proteins for degradation by the proteasome [56] ensuring that damaged, misfolded or unneeded proteins are efficiently removed. Indeed, proper functioning of ubiquitin mediated proteolysis (UPS) has been reported to be closely linked to the quality of early embryos with defects in this process suggested to lead to the accumulation of damaged or misfolded proteins, resulting in cellular stress and impaired development [57]. In addition, Ubiquitin-mediated proteolysis is known to tightly regulate the cell cycle by controlling the levels of key regulatory proteins such as cyclins, cyclin-dependent kinases (CDKs), and CDK inhibitors [58]. Finally, the ubiquitin-proteasome system (UPS) is involved in the regulation of cellular responses to stress [59] and regulates the levels of pro-apoptotic and anti-apoptotic factors, such as p53 and Bcl-2 family proteins [60]. But not only “ubiquitin mediated proteolysis” but also “Proteasome activity”, both acting as a tandem for the breakdown of protein was found to be among the highly affected pathways due to alterations of the developmental environment after maturation. The proteasome itself is a multi-subunit complex comprising a 20 S core particle and one or two 19 S regulatory particles. The 20 S core is responsible for the proteolytic degradation of substrates, while the 19 S regulatory particles recognize, unfold, and translocate ubiquitinated proteins into the core [56, 61]. The proteasome, which degrades misfolded or damaged proteins, has also been shown to play a key role in cell cycle progression, particularly during mitosis and meiosis, by targeting essential regulatory proteins such as cyclins and cyclin-dependent kinase inhibitors [62]. This process is critical for ensuring proper early embryonic cell divisions and preventing aneuploidy [63]. Thirdly, the present study revealed that genes differentially expressed particularly due to the environment after maturation play important roles in the “Cell cycle” pathway representing a crucial process in the early stages of embryonic development. This is in line with recent studies reporting correlations between the embryo transcriptome and morphokinetic of development [64, 65]. In addition, the in vitro environment as well as media composition [66] have been reported to affect developmental kinetics in embryos which has been associated with a morphokinetic-related response to stress recently [67]. Especially the dramatic down-regulation of Polo-Like Kinase 1 (PLK1) in bovine embryos developed within the in vitro environment after maturation attracted our attention. Previous reports have shown that PLK1 is critical for all stages of mitosis and one of the functions of PLK1 is to promote the G2/M transition by activation of the anaphase-promoting complex to facilitate the separation of sister chromatids [68]. Consequently, PLK1 is indispensable for the successful completion of cytokinesis [69, 70]. Since PLK1 homozygous null mice were demonstrated to be embryonic lethal [71], downregulation of PLK1 in bovine embryos due to the in vitro environment after maturation might therefore represent a clue for reduced vitalities of bovine embryos when cultured in vitro after maturation [2] as well as higher incidences for chromosomal aberrations [72].

### The sustained impact of the maturation environment on blastocyst transcriptome profile

To specifically asses the impact of the environment during in vitro maturation, we focused on DEGs identified between IFOT-M embryos and VIVO embryos that were not differentially expressed between IFOT-IM and VIVO embryos. The results of the present study revealed that DEGs specifically influenced by the in vitro environment during oocyte maturation play fundamental roles in key biological processes related to steroid metabolism including “steroid metabolic process” and “steroid biosynthetic process”. Accordingly, “Terpenoid backbone synthesis” and “steroid biosynthesis” emerged as the prominent pathways significantly affected by these DEGs, highlighting the sensitivity of steroidogenic pathways to the conditions present during maturation. To gain insights into the impact of these differentially regulated genes and the interactions among their corresponding proteins, we constructed a functional protein association network using STRING (*Search Tool for the Retrieval of Interacting).* In this regard, especially two genes involved in cholesterol synthesis attracted our attention, namely 3-Hydroxy-3-Methylglutaryl-CoA Synthase 1 (HMGCS1) known to be required for Isoprenoid and Cholesterol Synthesis and the endoplasmic reticulum enzyme 3-hydroxy-3-methylglutaryl coenzyme A (HMG-CoA) reductase known to catalyze the rate-limiting step in cholesterol biosynthesis. Studies with murine embryos homozygous for the HMG-CoA mutant allele revealed that HMG-CoA reductase is crucial for early development of the mouse embryos [73]. Interestingly, loss of HMG-CoA reductase activity did not affect developmental capacity up to the blastocyst stage but leads either to implantation failure or to embryonic death prior to implantation in murine [73] which is in accordance to the finding that cholesterol synthesis in embryos appears to start at the peri-implantation stage in mice [74, 75]. Furthermore, DHCR7 catalyzes the conversion of 7-dehydrocholesterol to cholesterol and DHCR24 catalyzes the reduction of the delta-24 double bond of sterol intermediates during cholesterol biosynthesis. Dysregulation of these genes due to the maturation environment is therefore in line with earlier studies reporting that the maturation environment affects the quality of bovine embryos due to its effects on the quantity of lipid droplets as well as their cryo-sensitivity. To that respect, down-regulation of HMGCS1, HMG-CoA, DHCR24 and DHCR7 might cumulatively reduce the quantity of cholesterol in bovine embryos. Keeping in mind that cholesterol is considered a major determinant for cryo-sensitivity of sperms [76] as well as oocytes [77] and embryos [78], down-regulation of these genes might explain higher cryo-sensitivity of in vitro derived bovine embryos [20, 22]. Of great impact, Stearoyl-CoA Desaturase 5 (SCD5) was identified to be the most connected protein among the DEGs due to the in vitro environment during maturation. SCD5 is an integral membrane protein of the endoplasmic reticulum that catalyzes the formation of monounsaturated fatty acids from saturated fatty acids via insertion of a cis double bond at the delta-9 position into fatty acyl-CoA substrates including palmitoyl-CoA and stearoyl-CoA. SCD is considered a key regulator of energy metabolism with a role in human obesity and dyslipidemia and SCD activity in cumulus cells effectively protects the oocyte against lipid-induced damage by conversion of saturated into monounsaturated fatty acids [79]. Keeping in mind that oleic acid prevents detrimental effects of saturated fatty acids on bovine oocyte developmental competence [79] as well as cryo-tolerance of subsequent embryos [80, 81], down-regulation of SCD5 due to the in vitro environment during maturation potentially resulting in lower amounts of oleic acid might be a clue for lower developmental capacity of in vitro matured bovine oocytes [4, 20] as well as lower cell numbers of subsequent blastocysts [82].

### The sustained impact of oocyte source on blastocyst transcriptome outline

To investigate the specific impact of the source of the oocytes, cumulus oocyte complexes collected from slaughterhouse ovaries were transferred immediately into a dominant follicle (IFOT-IM) allowing final in vivo maturation, in vivo fertilization as well as early embryo development in vivo and were compared with in vivo derived blastocyst (VIVO). DEGs of IFOT-IM embryos as a consequence of initial source of immature oocytes were found to play a fundamental role in “steroid biosynthesis”. This pathway is important for the production of the steroid hormones progesterone, estrogen, androgen and glucocorticoid along with their precursor cholesterol and play a role for proper embryo development and maintenance of a pregnancy in humans [83]. However, down-regulation of this pathway in IFOT-IM embryos compared to VIVO embryos, or alternatively up-regulation of this pathway in VIVO embryos compared to IFOT-IM embryos, may suggest that the hormonal superstimulation performed to obtain in vivo derived embryos influenced steroidogenesis in oocytes prior to final maturation, with effects persisting through to the blastocyst stage. Consequently, some DEGs between IFOT-IM and VIVO-derived embryos may not solely reflect differences in oocyte sources or differential selection, but could alternatively result from the effects of hormonal superstimulation. In particular, in vivo derived embryos generated by superovulation may have been exposed to elevated levels of progesterone and other hormones or steroids, in contrast to IFOT-IM embryos, which developed in unstimulated animals. Indeed, super-stimulation via the application of hormones has been reported to increase abnormal blastocyst formation and fetal growth retardation compared to those derived from non-stimulated mice [84]. Among these DEGs a total of 28 genes was shared between VITRO, IFOT-M and IFOT-IM embryos, respectively. Therefore, these DEGs represent highly confirmed candidates affected by the source of the ovaries. Remarkably, all 28 DEGs were found to be down-regulated in VITRO, IFOT-M and IFOT-IM compared to VIVO embryos. String interactome analysis revealed that dihydrolipoamide dehydrogenase (DLD) plays a prominent role among these DEGs. DLD is considered a multifunctional and therefore categorized as a moonlighting protein [85]. DLD catalyzes the oxidation of dihydrolipoyl moieties of four mitochondrial multienzyme complexes, namely pyruvate dehydrogenase complex (PDC), α-ketoglutarate dehydrogenase complex (KDC), branched-chain α-ketoacid dehydrogenase complex (BCKDC) and the glycine cleavage system GCS [86]. Among these, the PDH complex has been shown to play a pivotal role in oocyte meiotic maturation via its functions in catalyzing the conversion of pyruvates to acetyl-CoA [87]. Embryos carrying an inactivated murine pyruvate dehydrogenase gene were found to be globally delayed in development by 9.5 days post coitus, with resorption occurring over the following several days [86]. With regard to the KDC, it has been reported recently that Alpha-ketoglutarate plays an important role in murine embryo development through metabolic and epigenetic modulations [88]. Thus, we speculate that deficiencies in these two complexes might play a significant role in embryo lethality since absence of these two enzymatic activities would result in disability to metabolize glucose oxidatively. This outcome is in accordance to recent findings revealing lower oxygen consumption rates and therefore lower rates of oxidative phosphorylation of in vitro vs. in vivo derived embryos with even lower values in bovine IVP derived embryos of lower vitality [89]. Moreover, these insights may explain the generally accepted lower development rates and qualities of bovine IVP derived embryos compared to in vivo derived counterparts [1]. These observations in the mouse are in great agreement with an earlier study reporting absence of an embryonic disc in a majority of early elongation stage bovine embryos after transfer of IVP embryos [90] and is in line with downregulation of PLAC8 in all blastocyst groups derived from slaughterhouse ovaries. Keeping in mind that expression of PLAC8 has been reported to be positively correlated with calf delivery [91], these insights may explain lower pregnancy rates after transfer of bovine IVP derived embryos compared to in vivo derived counterparts [3].

Finally, the whole process of injecting oocytes into an ovarian follicle is technically complex and should not be underestimated, as it may itself influence gene expression. Therefore, the observed differences between VIVO and IFOT-IM embryos may not solely reflect differences in oocyte origin; they may also be partially attributed to the mechanical or physiological effects of the intrafollicular transfer procedure. An additional limitation relates to the selection of embryos based on the developmental stage used for transcriptomic analysis. Although great care was taken during embryo recovery, the possibility cannot be entirely excluded that some IFOT-M and IFOT-IM embryo pools also contained embryos that originated from the temporary recipient’s own oocytes, i.e. VIVO embryos. If this occurred, it would likely have reduced the observed differences between IFOT-M and IFOT-IM embryos compared to VIVO embryos. Consequently, the differences reported in this study are likely to be conservative, and the actual biological distinctions may be even more pronounced.

## Conclusion

Collectively, the results of the present study reveal for the first time the molecular mechanisms and pathways as distinct consequences of non-physiological environments during early embryonic development of bovine embryos. IVP derived embryos are characterized by dysregulation of genes playing a fundamental role in pathways involved in protein synthesis, protein degradation and cell-cycle regulation. Representing the most important findings, dysregulation of “ubiquitin mediated proteolysis”, “proteasome activity” as well as “cell cycle” pathways due to a non-physiological environment during fertilization and early embryo development might be a clue for failure of embryonic genome activation, precise DNA duplication and appropriate cell cleavage. Differential expression of distinct genes due to the non-physiological environment during maturation might explain alterations in terms of cholesterol synthesis and lipid metabolism determining cryo-tolerance of subsequent embryos. While intrafollicular transfer of immature oocytes into dominant follicles resulted in blastocysts closely resembling in vivo derived counterparts, our study also pointed out that the origin of the immature oocytes already predetermines the later embryo quality implicating a compromised capability for post-implantation development. However, the interpretation of the data may be partly influenced by the fact that VIVO embryos are obtained after superstimulation, whereas IFOT-IM embryos are obtained from single ovulated follicles. Taken all together, the present study thus provides a comprehensive inventory of the expression outline of relevant transcripts and pathways as a consequence of the respective developmental environments. Taking advantage of this inventory is expected to be useful for further studies aiming to enlighten our understanding with regard to the attainment of embryonic developmental capacity and improving embryo in vitro culture conditions.

## Supplementary Information

Supplementary Material 1.

Supplementary Material 2.

Supplementary Material 3.

Supplementary Material 4.

Supplementary Material 5.

Supplementary Material 6.

Supplementary Material 7.

Supplementary Material 8.

Supplementary Material 9.

Supplementary Material 10.

## Data Availability

The dataset used and/or analyzed during the present study were deposited into the Gene Expression Omnibus database under accession number GSE284466 and are available at the following URL: https://www.ncbi.nlm.nih.gov/geo/query/acc.cgi? acc=GSE284466.

## Abbreviations

VIVO: In vivo
VITRO: In vitro
IFOT-M: Intrafollicular oocyte transfer of in vitro matured oocyte
IFOT-IM: Intrafollicular oocyte transfer of immature oocyte
BSA: Bovine serum albumin
FBS: Fetal bovine serum
ECS: Estrous cow serum
GnRH: Gonadotrophin releasing hormone
PBS: Phosphate buffered saline
PCA: Principal component analysis
TAC: Transcription analysis console
DEG: Differentially expressed genes
FDR: False discovery ratio
FC: Fold change
GO: Gene ontology
