# Supplementary figures and images for: Comparative transcriptome analysis of bovine blastocysts reveals specific effects of the oocyte source and the environments during maturation and early embryo development
Source: BMC Genomics. 2025 Jul 17;26:673. doi: 10.1186/s12864-025-11848-8 (PMC12273470; doi:10.1186/s12864-025-11848-8)

Supplemental Figure 1, Hoelker et al.

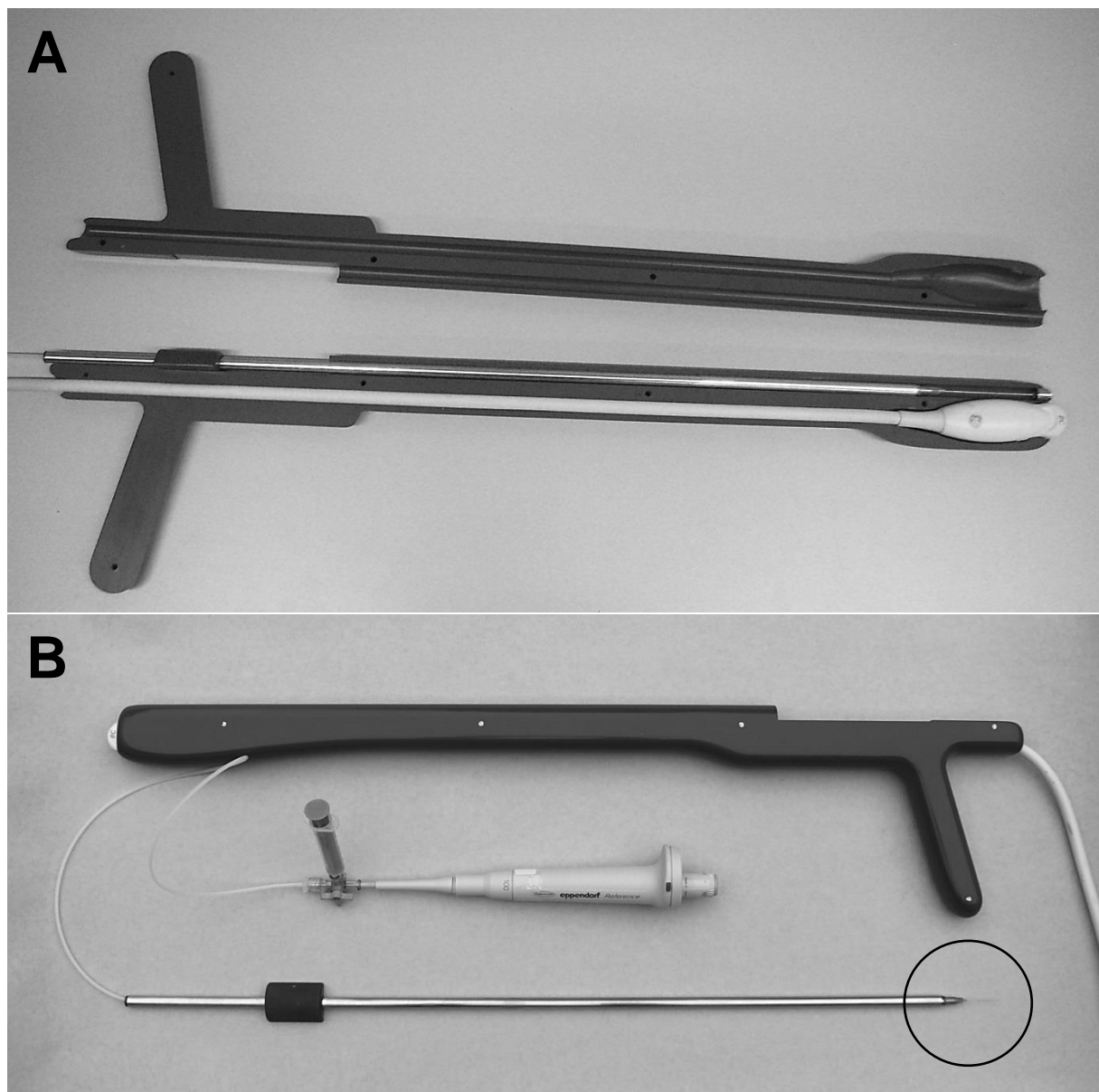

Supplement: Supplementary file 1 — Supplementary Material 1. [file 12864_2025_11848_MOESM1_ESM.pdf]

Supplemental Figure 1, Hoelker et al.

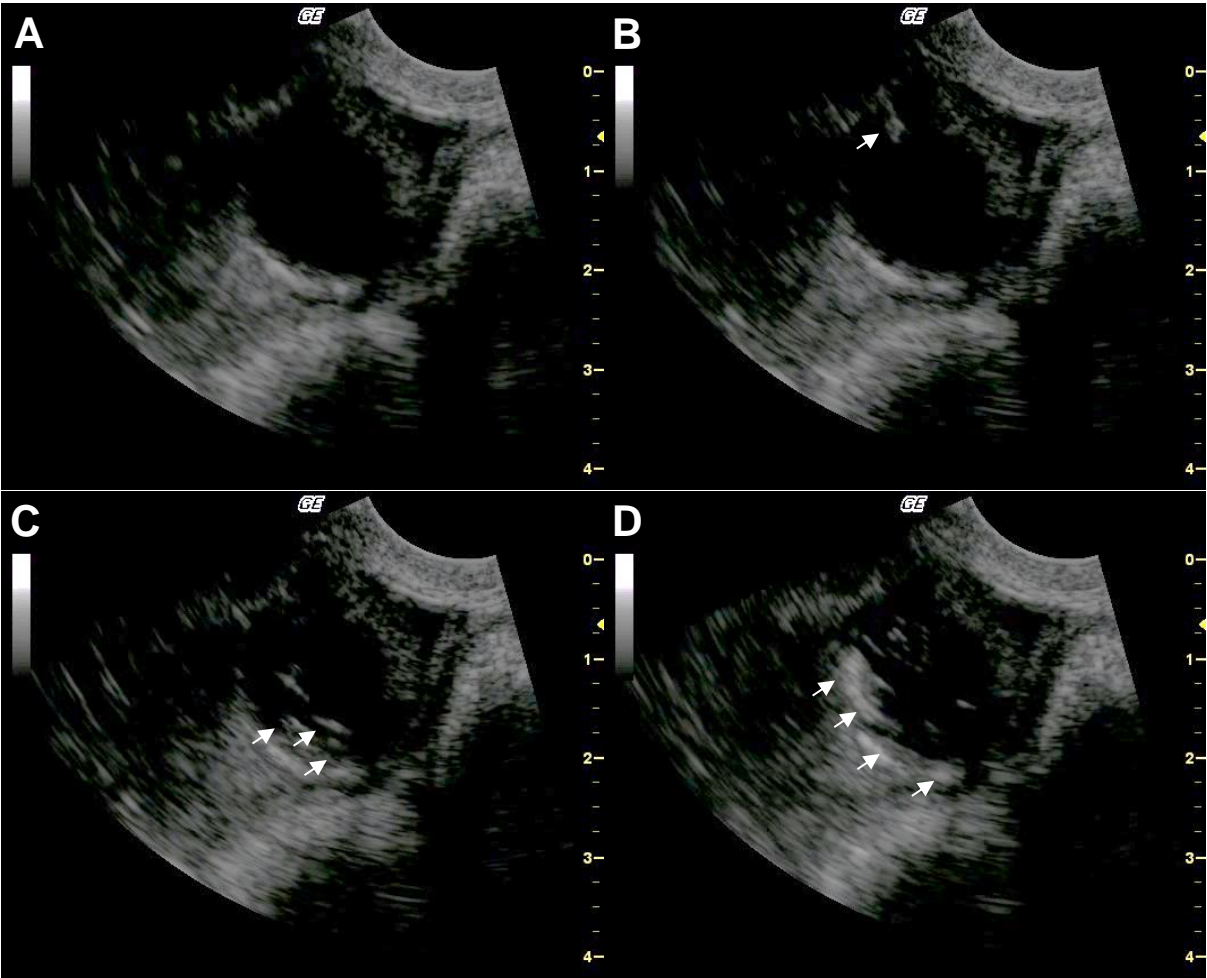

Supplement: Supplementary file 2 — Supplementary Material 2. [file 12864_2025_11848_MOESM2_ESM.pdf]

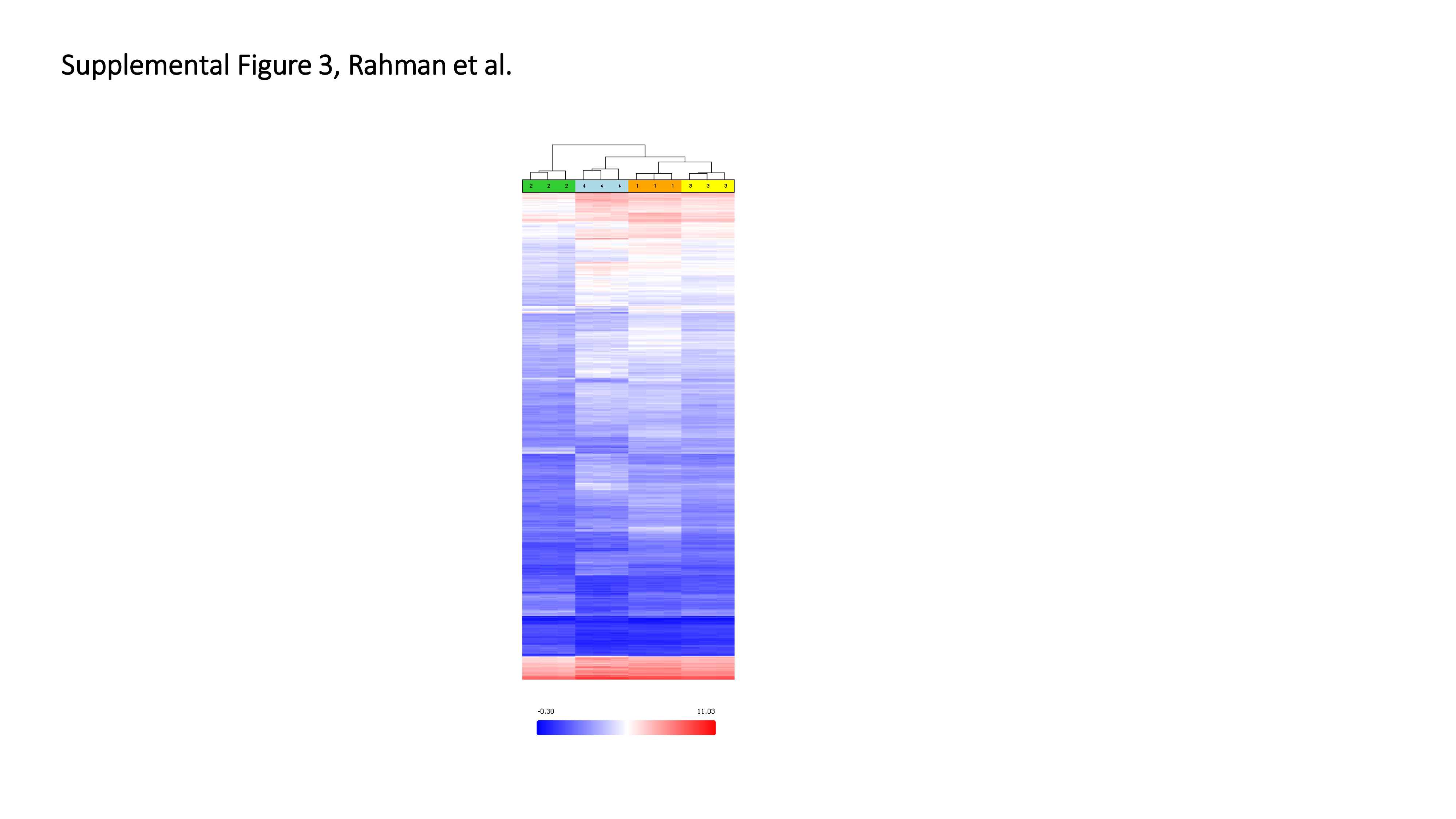

Supplement: Supplementary file 10 — Supplementary Material 10. [file 12864_2025_11848_MOESM10_ESM.tif]
